# Supplementary material for: Evolutionary characteristics of global offshore carbon emissions network and responsibility allocation of emissions reduction
Source: Patterns (N Y). 2023 Jul 31;4(10):100801. doi: 10.1016/j.patter.2023.100801 (PMC10591139; doi:10.1016/j.patter.2023.100801)
Supplement: Document S1. Figure S1 and Tables S1–S4 [file mmc1.pdf]

Patterns, Volume *4*

Supplemental information

Evolutionary characteristics of global offshore carbon emissions network and responsibility allocation of emissions reduction

Bo Lu, Yue Sun, Lijie Fan, Xuejiao Ma, and Hongbo Duan

Supplementary Information for

Evolutionary characteristics of global offshore carbon emissions network and responsibility allocation of emissions reduction

Bo Lu [1,*,#], Yue Sun [1], Lijie Fan [1], Xuejiao Ma [1,*], Hongbo Duan [2,*]

[1] School of Economics and Management, Dalian University of Technology, Dalian, 116024, China
[2] School of Economics and Management, University of Chinese Academy of Sciences, Beijing, 100190, China
* Correspondence: Email: lubo@dlut.edu.cn; maxuejiao@dlut.edu.cn; hbduan@ucas.ac.cn
[#] Lead Contact: Email: lubo@dlut.edu.cn

This PDF file includes:

Figure S1

Table S1-S4

Figure S1

Global offshore carbon emissions from 2015 to 2021 show a tendency of decrease firstly and then increase from 21112.58 Kt in 2015 to19360.38 Kt in 2018 to 20581.57 Kt in 2021 as shown in **Figure S1**. This may be because IMO has implemented the *Ship Energy Efficiency Management Plan* in 2014 to reduce energy efficiency, substitute traditional fuels by liquefied natural gas and alternative fuels and conduct operational mitigation measures. After 2018, the globalization accelerates the greenhouse gas emissions by promoting the continuous demands for sea transportation, causing the rise of carbon emissions. Therefore, there is a close relationship among the global trade, shipping and carbon emissions.

Carbon emissions from ports of top 20 countries contribute to about 80% of total port carbon emissions in the world, and top 20 countries are displayed in **Figure S1**. Among the 20 countries, there are 12 developed countries that generate 48% of port carbon emissions; while the other 8 developing countries generate about 52% of port carbon emissions. They are mainly located in Asia such as China and Singapore and Europe such as Italy and Spain. Specifically, China generates the largest carbon emissions from ports each year, followed by the U.S. and Singapore. As the biggest developing country, it has huge import and export markets, and the frequent seaborne trade is the root cause of its high port carbon emissions. The top 10 list of global port container throughput in 2021 includes 7 Chinese ports, and Shanghai port has always been in the top 1 position with nearly 10 million TEU more than the second port of Alphaliner. The industrial consumer goods of the U.S. are mainly dependent on imports, and its Log Angeles and Long Beach, with superior geography position, concentrate on external transportation, forming a commercial transportation system. So the intensive navigation of ships leads to the output of a very large amount of carbon emissions from ports. The Singapore Port has maintained its status as a global hub port and international maritime center. With the development of economic globalization, the increasing demands for shipping services have boosted the increase of carbon emissions from ports.

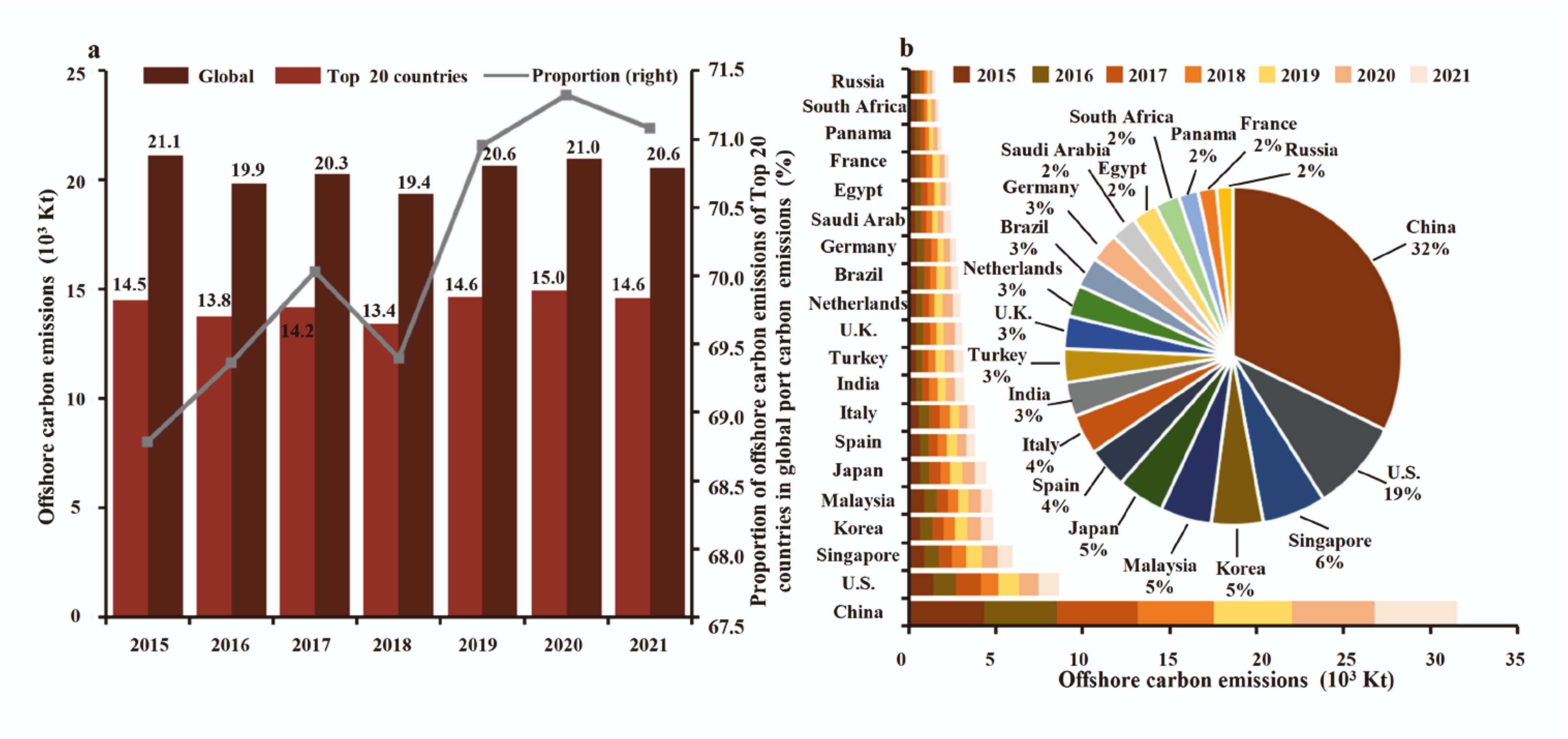

Figure S1. Offshore carbon emissions during 2015 and 2021

a, Total carbon emissions from global offshore and top 20 countries, and the proportion of offshore carbon emissions from top 20 offshore countries to global total offshore carbon emissions from 2015 to 2021. b, Evolution of offshore carbon emissions in top 20 countries during 2015 and 2021 (The pie chart shows the share of the average carbon emissions of top 20 countries from 2015 to 2021

Table S1. The developed countries and economies defined as the Annex I Parties of the Kyoto Protocol[1].

Table S1. The developed countries and economies defined as the Annex I Parties of the Kyoto Protocol.

**Developed countries and economies**		
United States of America	Germany	New Zealand
Australia	Greece	Norway
The Republic of Austria	Hungary	Poland
Belgium	Iceland	Portugal
Bulgaria	Ireland	Romania
Canada	Italy	Russian Federation
Croatia	Japan	The Slovak Republic
The Czech Republic	Latvia	Slovenia
Denmark	Liechtenstein	Spain
Estonia	Lithuania	Sweden
Finland	Luxembourg	Switzerland
European Community	Monaco	Ukraine
France	Netherlands	The United Kingdom of Great Britain and Northern Ireland

Table S2. Parameter values of engine's installed power, average cruising speed and average maximum design speed for container ships with different load.

This table illustrates average propulsion engine's installed power ($ME_{install,w}$), average cruising speed ($V_{man,w}$) and average maximum design speed ($V_{design,w}$) applied in this study for global container shipping emissions and energy consumption estimation.

Table S2. Parameter values of engine's installed power, average cruising speed and average maximum design speed for container ships with different load

**Size category**	**2015** $ME_{install}$ **(kW)**	**2015** V_{design} **(kn)**	**2015** V_{man} **(kn)**	**2016** $ME_{install}$ **(kW)**	**2016** V_{design} **(kn)**	**2016** V_{man} **(kn)**	**2017** $ME_{install}$ **(kW)**	**2017** V_{design} **(kn)**	**2017** V_{man} **(kn)**	**2018-2020** $ME_{install}$ **(kW)**	**2018-2020** V_{design} **(kn)**	**2018-2020** V_{man} **(kn)**
0-999 TEU	5633	16.2	12.2	5509	16.1	12.2	5324	16.1	12.1	5077	16.0	11.8
1000-1999 TEU	12160	19.0	13.6	12119	19.0	13.7	12093	19.0	13.6	12083	19.0	13.4
2000-2999 TEU	21341	21.3	14.0	21010	21.2	14.1	20851	21.2	14.2	20630	21.1	14.2
3000-4999 TEU	35082	23.2	14.8	35133	23.2	14.8	34782	23.2	14.7	34559	23.1	14.7
5000-7999 TEU	52646	24.6	15.4	52597	24.6	15.5	52584	24.6	15.9	52566	24.6	15.7
8000-11999 TEU	59597	24.2	15.7	58665	24.0	16.1	58143	24.0	16.4	57901	23.9	16.3
12000-14499 TEU	65348	24	16.0	64498	23.9	16.3	62923	23.8	16.4	61231	23.8	16.3
14500-19999 TEU	61829	20.3	16.9	61417	20.0	17.1	60532	20.1	16.8	60202	20.2	16.5
20000-+ TEU	N/A	N/A	N/A	N/A	N/A	N/A	60681	20.4	15.2	60210	20.3	16.3

Table S3. Calculation of network characteristics

Table S3. Indicators of overall network and individual countries

Property	Name	Meaning	Equation	Practical implications	Value
Overall network	Network correlation	Direct or indirect path of connection between any two nodes	$NC = 1 - \frac{V}{N \times (N-1)/2}$	It reflects the stability of the network.	Higher value means a more stable network
	Network hierarchy	Asymmetrical reachability between nodes	$NH = 1 - \frac{K}{\max(K)}$	It reflects the dominant and hierarchical position of each country, reflecting the overall degree of asymmetry and whether each country plays an equal role.	Higher value means stricter hierarchical structure, and offshore carbon emissions between some countries are in a monopolic position.
	Network efficiency	Redundant connections between nodes	$NE = 1 - \frac{M}{\max(\mathrm{M})}$	It reflects the number of links between countries and channels for receiving and spilling carbon emissions.	Higher value means offshore carbon emissions have limited transfer channels.
Individual network	Degree centrality	The number of nodes directly related to a node	$DC(i) = \frac{n_{in} + n_{iout}}{2(N-1)}$	It reflects the status and control power of a country and whether a country is in a central position.	Higher value means important role and greater influence on other countries.
	Betweenness centrality	The number of the shortest paths of a node between any two other nodes	$C_B(i) = \frac{\sum_{j=1}^{N-1}\sum_{k=2}^{N} b_{jk}(i)}{(N^2 - 3N + 2)/2}$	It reflects the extent to which a country is able to act as an intermediary and the ability to control the transmission of information.	Higher value means stronger ability to control the relationship between other countries.

Note: *V* is the number of unreachable paired points in the network; *N* is the number of nodes in the network; $N \times (N-1)/2$ indicates the maximum number of possible connections in the network; *K* is the number of symmetrically reachable paired nodes in the network; max(*K*) is the maximum number of symmetrically reachable paired nodes in the network[2]; *M* is the number of extra

connections in the network; max(M) is the maximum number of possible redundant connections in the network; n_{in} represents the entry degree of the node (country) i; n_{iout} represents the output degree of the node (country) i; ($n_{in}+n_{iout}$) represents the number of countries directly connected to country i; $b_{jk}(i)$ represents the probability that country i lies between country j and k; $(N^2-3N+2)/2$ represents the maximum possible value of the betweenness centrality of a country in the case of star networks[3].

Table S4. Principal of block model

First, bidirectional spillover plate has many correlations with other plates and there are also close correlations between countries within the plate. Countries in this plate usually have the biggest ports in the world with close shipping trade with other countries, generating more carbon emissions. They play a leading role in the offshore carbon emissions network, and have a key driving effect on the other countries. Second, the broker plate is not closely connected with other plates, and connections between countries within it is few as well, which plays an intermediary role in the overall network. Countries in this plate can link different countries and ports may be in transit positions in global shipping trade. Third, in the main inflow plate, offshore carbon relationships between countries mainly occur within it, and there are less relationships with countries in other plates. Most of carbon emissions are from other plates, indicating that countries in this plate are the receivers of offshore carbon emissions. Fourth, in the main outflow plate, countries have more relationships with countries in other plates instead of countries within the plate. Most of the offshore carbon emissions of countries in this plate flow out and transfer to countries in other plates.

Table S4. Classification of offshore carbon emissions plate attributes of block model

Proportion of relations within plate	Proportion of relations received by plate	
	≈0	>0
$\geq (g-1)/(N-1)$	Bidirectional spillover plate	Main inflow plate
$< (g-1)/(N-1)$	Main outflow plate	Broker plate

Note: *g* is the number of members within the plate; *N* is the number of members in the whole network; (*g*-1)/(*n*-1) represents the expected internal relationship ratio. The total number of relationships received (sent) from (to) the plate is defined as the sum of the number of relationships for each column (row) in the received relationships matrix. Proportion of relations within the plate is the number of relationships in the plate divided by the total number of plate overflow relationships. Proportion of relations received by the plate is the number of relationships received from the other plates divided by the number of relationships sent to the other plates. When proportion of relations within plate is larger than the expected internal relationship ratio, it indicates that there is a closer correlation between the members within the plate. When proportion of relations received by plate is close to 0, indicating that there is a closer correlation between this plate and other plates.

References

1. Nations, U. (1998). Kyoto Protocol.

2. John Scott (2007). Social Network Analysis: A Handbook (Sage Publication).

3. Linton C. Freeman (1977). A Set of Measures of Centrality Based on Betweenness (Sociometry).
