# Supplementary material for: Evolutionary characteristics of global offshore carbon emissions network and responsibility allocation of emissions reduction
Source: Patterns (N Y). 2023 Jul 31;4(10):100801. doi: 10.1016/j.patter.2023.100801 (PMC10591139; doi:10.1016/j.patter.2023.100801)
Supplement: Document S2. Article plus supplemental information [file mmc2.pdf]

Article

Patterns

Evolutionary characteristics of global offshore carbon emissions network and responsibility allocation of emissions reduction

Highlights

- Global offshore carbon emissions present a complicated network structure
- The network is dominated by developed countries and large economies
- Similar countries have closer and more frequent carbon correlations
- Greater responsibilities should be allocated to countries at the center of the network

Authors

Bo Lu, Yue Sun, Lijie Fan, Xuejiao Ma, Hongbo Duan

Correspondence

lubo@dlut.edu.cn (B.L.),
maxuejiao@dlut.edu.cn (X.M.),
hbduan@ucas.ac.cn (H.D.)

In brief

Current studies mainly regard offshore carbon emissions from the shipping trade as independent, without considering the spatial correlation brought by trade networks. Thus, the responsibility allocation of emissions reduction is directly based on the emission quantity, not relative position. In fact, it shows a complicated network topology and spatial pattern, and it is dominated by developed countries and large economies, which can be used as a basis for responsibility allocation for emissions reduction, taking into account both fairness and efficiency.

Lu et al., 2023, Patterns 4, 100801
October 13, 2023 © 2023 The Authors.
https://doi.org/10.1016/j.patter.2023.100801

CellPress

Patterns

CellPress
OPEN ACCESS

Article

Evolutionary characteristics of global offshore carbon emissions network and responsibility allocation of emissions reduction

Bo Lu,[1,3,*] Yue Sun,[1] Lijie Fan,[1] Xuejiao Ma,[1,*] and Hongbo Duan[2,*]
[1]School of Economics and Management, Dalian University of Technology, Dalian 116024, China
[2]School of Economics and Management, University of Chinese Academy of Sciences, Beijing 100190, China
[3]Lead contact
*Correspondence: lubo@dlut.edu.cn (B.L.), maxuejiao@dlut.edu.cn (X.M.), hbduan@ucas.ac.cn (H.D.)
https://doi.org/10.1016/j.patter.2023.100801

THE BIGGER PICTURE The global reduction of emissions from the shipping trade is an important issue of sustainable development. Offshore emissions, as a component that can be captured and easily classified as responsible entities, exhibit network characteristics due to trade linkages. Therefore, we construct offshore carbon emission networks based on container shipping data to determine three things: first, the positions and roles of different regions and countries in the network and how they are related; second, leading and controlling functions in these easily captured shipping trade emissions and their impacts on other entities; and finally, efficient and clear allocation of relative responsibility for carbon reduction based on network structure and participants' positions. The positioning and correlation in the network system are the extensions for clearly allocating reduction responsibility, considering both fairness and efficiency.

1 2 3 4 5 **Proof-of-Concept:** Data science output has been formulated, implemented, and tested for one domain/problem

SUMMARY

Offshore carbon emissions from the international shipping trade are significant contributors to climate change. Based on the complex shipping trade networks, offshore carbon emissions are correlated rather than independent, and allocating responsibility for reducing emissions does not depend solely on the amount but on linkages. We use the global container shipping data covering more than 98% of routes from 2015 to 2020 to calculate the offshore carbon emissions from shipping. Subsequently, we construct an offshore carbon emissions network based on the shipping routes and emissions to identify the evolutionary tendency of network and clarify emissions reduction responsibilities by considering equity and efficiency. We discover that global offshore carbon emissions present a complicated network structure dominated by developed countries and large economies. Countries on the same continent or within the same economic organizations have closer and more frequent carbon correlations. Greater responsibilities should be allocated to countries who are at the center of the network.

INTRODUCTION

Shipping plays a pivotal role in the international transportation of import and export commodities, representing more than 70% of the global trade volume of goods completed by shipping.[1,2] International shipping accounts for about 3% of global greenhouse gas (GHG) emissions,[3–5] equivalent to the emissions of the sixth largest emitting country. Because of the continuous growth of global shipping trade, emissions from international shipping are likely to rise 40% by 2050 on a 2008 basis level.[3] Therefore, the International Maritime Organization (IMO) has set clear goals to decrease total carbon emissions by 50% by 2050 compared with 2008 and a more optimistic goal of shipping net-zero GHG emissions at the same time horizon[6]; however, continued growth in international trade and maritime activity also portends pressure for net-zero carbon emissions. Such concerns have prompted studies on new low-carbon energy production and storage,[7] and it could consider a new international cooperation framework by mobilizing the initiative of stakeholders[8] to stimulate net-zero shipping carbon.

This is an open access article under the CC BY license (http://creativecommons.org/licenses/by/4.0/).

As an important part of shipping, offshore carbon emissions are the discharge of ships; these emissions do not exist independently,[9] and different shipping lines connect them. Especially with the increasing frequency of shipping trade, the spatial correlation of shipping offshore carbon emissions has become increasingly complex and exceeded the simple geographical neighbor relationship, presenting network characteristics.[10,11] Among large economies with various receiving and spilling carbon emissions channels, in particular, the spatial correlation characteristics of carbon emissions are more widespread and extensive.[12–15] The spatial structural characteristics of global offshore carbon emissions should be investigated to define offshore carbon emissions worldwide.

Therefore, the responsibility for offshore carbon emission reduction can be identified more accurately.[14] Existing research on the allocation of carbon emission reduction responsibility focuses on the principle of equity and efficiency.[16–19] The equity principle means that each country should have the same carbon emission rights, and countries with high historical carbon emissions should reduce their carbon emission rights in the future.[18,20,21] The efficiency principle represents the coordinated development of the economy and environment, which maximizes economic output and optimizes resource allocation in the given carbon emission space.[21,22] If the carbon correlation is ignored, a mismatch remains between the responsibility allocation and the actual role of carbon emissions[23]; thus, it is challenging to balance fairness and efficiency. In this context, it is important to accurately reveal the roles of each country in the global offshore carbon emissions network and depict their spatial correlations. For example, implement common but differentiated responsibilities for developed and developing countries. The developed countries are countries with high economic, technological, educational, social welfare, and living standards, defined as the Annex I Parties of the Kyoto Protocol in the emission field,[24] while the developing countries are the reverse (see Table S1 for a detailed list).[25,26] Such insights can provide significant references for making cross-regional collaborative policies to mitigate offshore carbon emissions and allocating responsibilities of carbon emissions reduction.[27]

Current studies regard marine or offshore carbon emissions independently without considering the spatial correlations.[2,28,29] Furthermore, in the regional and industrial economy field, some recent literature has identified the spatial structure characteristics of carbon emissions networks[9,30]; however, few have focused on the shipping and offshore ports. Two types of methods can be applied to capture the spatial correlations of carbon emissions: one belongs to spatial econometric models,[14,31] and the other is the network method.[9,15] The former method highly depends on the attribute data that are the absolute value of carbon emissions, which cannot reveal the spatial association based on structural relationships. The network method is superior in depicting the multiple channels of carbon correlation and attracts greater attention from scholars.[32,33] Regarding the responsibility allocation of carbon emissions reduction, linear and non-linear optimal allocation models are applied.[16,34] As a typical linear programming model, the data envelopment analysis is usually used,[35–37] including different types.[38–40] Non-linear optimization models mainly include cooperative game theories,[41] systematic and quantitative methods,[42] computable general equilibrium mode,[43] multi-source data Bayesian methods,[44] influential node detection algorithm,[45] and particle swarm optimization (PSO) algorithms.[23] Among them, the swarm intelligence optimization algorithm is superior in solving the non-linear problem by imitating the foraging behavior of birds with good robustness. Moreover, we could improve the PSO algorithm by considering equity and efficiency based on the constructed offshore carbon emissions network. The carbon emissions generated by the shipping trade and the weight of the carbon emission network among various countries are selected as indicators of emission reduction capacity, responsibility, and potential.[4]

Generally speaking, current studies fail to discuss offshore carbon emissions from the network perspective or to allocate emissions responsibilities based on the established network. For the above research gaps, this study aims to construct an offshore carbon emissions network based on container shipping data to reflect the evolutionary tendency and spatial correlations of offshore carbon emissions. Based on the role each country plays in the established network, the responsibilities of reducing carbon emissions are clarified by applying the artificial intelligence optimization algorithm. Specifically, our study makes the following contributions. First, unlike most relevant literature that only measures emissions in the global liner shipping trade, this paper further clarifies the spatial relationship of carbon emissions in the shipping process and deconstructs the distribution, correlation, and evolutionary characteristics of each country (ports) in the offshore carbon emissions network. We innovatively establish a global offshore carbon emissions network using the records of about 1,700 container shipping lines that cover 98% of global container shipping lines to uncover the spatial structure and evolutionary characteristics of global offshore carbon emission more visually. Based on the modified gravity model and social network analysis, we capture the connected feature of the offshore carbon emissions network from the whole to the individual. Second, it breaks the limitation of emission responsibility allocation in the existing literature; we apply the modified PSO algorithm to allocate responsibilities for offshore carbon emissions reduction with the target of reducing by 5%. Since previous studies mainly calculate that fairness and efficiency are hard without considering the relevance of carbon emission, we consider the role and carbon correlation of each country in the offshore carbon emissions network rather than the direct emissions, which makes the responsibility allocation fairer. Moreover, we allocate larger weights to countries whose role in the network is more central in reducing carbon emissions efficiently, since major economies and developed countries with advanced international shipping trade should take primary responsibility for offshore carbon emissions. Thus, this work provides insights into the evolutionary tendency of offshore carbon emissions networks. More importantly, it paves the way for more advanced studies on the collaborative reduction in carbon emissions and assignment of responsibilities.

RESULTS

Spatial-temporal evolution of offshore carbon emissions

The offshore carbon emissions results are provided in Figure S1, and we analyzed in detail the trend of offshore carbon emissions by countries recently; more details are presented in the supplemental information. In the offshore carbon emissions network,

CellPress
OPEN ACCESS

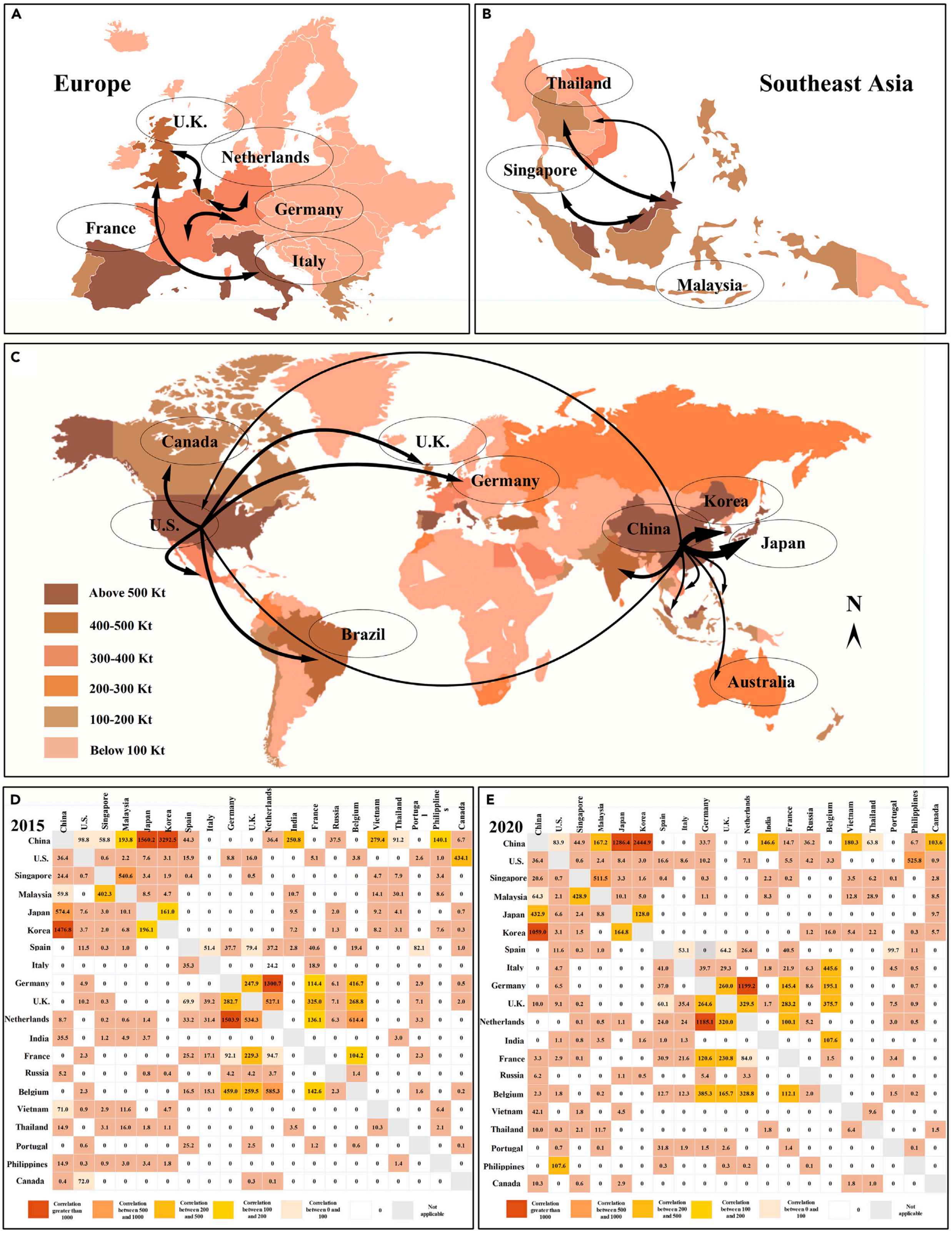

Figure 1. Offshore carbon emissions correlation between countries

(A) Offshore carbon emissions correlation between European countries, including the UK, Germany, Italy, France, and the Netherlands.

(B) Offshore carbon emissions correlation between countries within Southeast Asia, including Malaysia, Thailand, and Singapore.

(legend continued on next page)

the connection relationship and direction of the network are constructed based on the container shipping trade data; thus, the connections of the offshore carbon emissions network can represent the carbon correlation from one country to another. International trade and geographical location mainly affect the offshore carbon correlation between countries. Figure 1 presents the carbon correlation among the top 20 countries measured by the modified gravity model. From the overall perspective, countries with large offshore carbon correlation are more likely to be on the same continent owing to the short geographical distance and convenient port traffic. Among them, carbon correlation within Asia is the largest, centered on China and Singapore, with two-way exchanges with other countries, including South Korea, Japan, India, and Malaysia. In Asia, 353 ports generated the most significant carbon emissions over the years, and in 2020 carbon emissions from Asia ports reached 11,843.90 kt. The carbon correlation between China and Korea is far more than that of other countries, with an average annual growth of about 6.23% in recent years, because their geographical location is similar, and the shipping trade is close since China has been the largest trading partner of Korea for many years. The carbon correlation between Singapore and Malaysia ranks in the top 10 globally for similar reasons to China and Korea.

The number of ports in Europe is less than that in Asia, and carbon emissions in 2020 were 4,170.40 kt, forming an internal association network centered on the European Union (EU) countries. The carbon correlation of developed countries such as Germany, France, Italy, and the Netherlands is far greater than that of other countries. Affected by the EU economies, foreign trade greatly influences the entire shipping trade through large ports such as Rotterdam, Antwerp, Bremen, and Hamburg. The carbon correlation in North America is mainly caused by the trade between the US and Canada; in fact, the US transfers more carbon emissions to Canada. This result may be because the US is adjacent to Canada, and its container throughput ranks second only to China. In particular, the carbon correlation between Africa and South America is relatively small, resulting from the degree of economic development.

From the cross-regional perspective, the offshore carbon emissions correlation between China and the US is the largest since they are both big countries for the container shipping trade, indicating their strong carbon emissions association. The representative carbon correlation in the network is the connection between China, the EU, and the US, which also confirms the close relationships between trade and carbon emissions. Because of geographical location, industrial structure, and resource demands, carbon correlation mainly evolves around developed countries or major economies that play a pivotal role in the network structure. In comparison, some developing countries with backward economic development are carbon receivers. For example, although South Africa is the most developed country in Africa, it receives carbon along the shipping trade from the US, Singapore, Brazil, and India.

Characteristics of offshore carbon emissions network

The spatial correlation of global offshore carbon emissions is not limited to the neighbor relationship in the geographical sense, instead demonstrating a directed complex network structure. Figure 2 shows that the average of the network correlation approximates 1, implying a steady development trend with few isolated countries. The mean value of the network hierarchy is only 0.053, indicating that the carbon correlation between countries is bidirectional and spatially related. The relatively strict spatial correlation hierarchy in the overall carbon emissions network is gradually broken, enhancing the interaction. The network efficiency tends to be 0.926, which indicates that channels for receiving and spilling carbon correlation of countries are limited, and a small group of countries that produce significant carbon emissions dominate the offshore carbon emissions network.

The evolution tendency of the overall characteristics of the global offshore carbon emissions network reveals that, with the development of economic globalization, the closer relationship between countries directly leads to a more complicated carbon flow network. Owing to the unbalanced development of regional trade, the carbon correlation between countries in the network is clear, as presented in Figures 2A–2C. Countries such as China, Japan, Korea, the US, and Singapore occupy the most important positions, while Argentina, Peru, and Norway rank second, and Greenland, Tulua, and Cape Verde play the least important role in the network. Associations in the offshore carbon emissions network concentrate on major core economies closely related to the port trade.

The degree centrality and betweenness centrality indicators are calculated to reveal the role of each country in the offshore carbon emissions network. The degree centrality measures the number of countries directly connected with the country regarding carbon correlation, and Figures 2D–2F present the results. In the global offshore carbon emissions network, the average degree centrality increases from 2015 to 2020, and there are more than 80 countries whose degree centrality is higher than the average, with a proportion of nearly 52.98%, indicating more connections with other countries. Of the top 20 countries with degree centrality, 12 belong to developed countries, and the others are emerging economies. Among them, China ranks first, followed by the US. The positions of Singapore, Spain, Germany, and Italy maintain stability, while the centrality of some developing countries, such as Vietnam, rises quickly owing to trade development. Countries whose degree centrality is approximately zero, such as Tulu and Tango, have almost no spatial connections with other countries and occupy an edge position. The betweenness centrality reflects the degree to which a country can affect the connections between other countries, and countries with higher betweenness centrality are more active in the global offshore carbon emissions network, as shown in Figures 2G–2I. There are 34 countries whose betweenness centrality is higher than the average value. Among them are 13 Asian countries that China, Singapore, and Malaysia represent, and nine countries belonging to Europe, such as Spain and the UK.

(C) Global offshore carbon emissions correlation between countries including the US, Canada, Brazil, the UK, Germany, China, Korea, Japan, and Australia.

(D) Offshore carbon emissions correlation of top 20 countries in 2015.

(E) Offshore carbon emissions correlation of top 20 countries in 2020.

CellPress
OPEN ACCESS

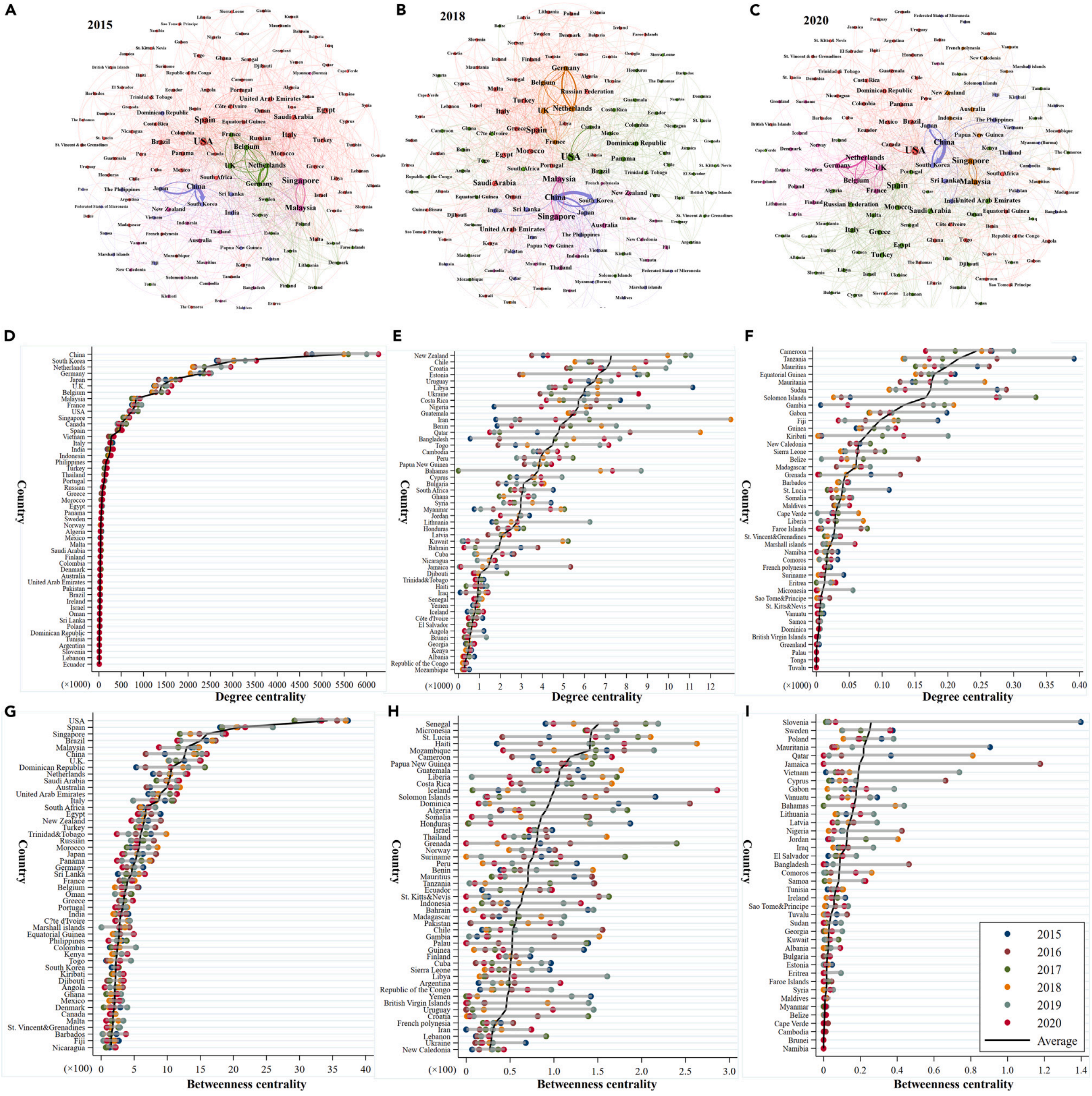

Figure 2. Global offshore carbon emissions network and centrality characteristics

(A) Global offshore carbon emissions network structure in 2015; 153 countries are involved in the network, and the thickness of the line indicates the weight of carbon correlation between countries. Block colors are divided according to the community detection algorithm, and communities with the same color are nodes with the same characteristics.

(B) Global offshore carbon emissions network structure in 2018.

(C) Global offshore carbon emissions network structure in 2020.

(D) Degree centrality of top 50 countries from 2015 to 2020.

(E) Degree centrality of top 51–100 countries from 2015 to 2020.

(F) Degree centrality of top 101–142 countries from 2015 to 2020.

(G) Betweenness centrality of top 50 countries from 2015 to 2020.

(H) Betweenness centrality of top 51–100 countries from 2015 to 2020.

(I) Betweenness centrality of top 101–140 countries from 2015 to 2020.

The US has the highest betweenness centrality and can control and dominate other countries in the offshore carbon emissions network. Other countries control nations such as Uruguay, Cambodia, and Namibia, which depend on those dominant countries.

It is worth mentioning that the degree and betweenness centrality of the network reflect the different roles of states in the network. The former reflects the status and importance of a country, while the latter shows the connectivity and control ability of a country acting as a bridge. Owing to frequent trade, some countries contribute a lot to offshore emissions, but their control over the entire network is not robust, and they could hinder the transfer of offshore carbon emissions, such as Sweden and Cambodia. China is the most important of the top two states but is less controlled than the United States. The USA is a busy broker, effectively controlling the network, and its extensive trade links facilitate the transfer of offshore emissions across the network.

Analyses of the centrality features imply that the spatial correlation of the global offshore carbon emissions network shows a pronounced Matthew effect. Developed countries and leading economies play a central role with powerful controlling abilities over other countries. In comparison, less developed countries have significant gaps and occupy the edging position. The centrality indicators of the offshore carbon emissions network are imbalanced, and the network evolution tendency lies in the correlation of core countries.

Core-edge analysis of the offshore carbon emissions network

The core-edge analysis is used to reveal the position of different countries in the offshore carbon emissions network. As shown in Figure 3, the core-edge structure existed during 2015 and 2020 with the standardized coreness of above 75% economies less than 0.1. The coreness of the top 50 countries (Figure 3A) in the network exceeds 0.06. These countries occupy a relatively important position, including all core countries (29 countries) and some semi-edged countries. Most of these countries are located on the vital shipping lines of Western Europe, North America, and East and Southeast Asia, with frequent maritime trade between countries and high port transfer rates and throughput. For the top 51–100 countries (Figure 3B), their coreness is between 0.01 and 0.06, including only three edged countries; the rest are semi-edged countries. Most of these countries are located in Africa and Southwest Asia and have been actively participating in maritime trade and developing their economies in recent years. The last 52 countries (Figure 3C), whose coreness is less than 0.01, are almost all edged countries with small and island economies at the most marginal position of the whole network.

In detail, for specific countries in the offshore carbon emissions network, the core of the US, Spain, and Singapore rank the top three and play the most critical role. The coreness of the US experiences a U shape, decreasing to 0.276 in 2018 and rapidly increasing to 0.291 in 2020. There is no significant change in the coreness of Spain, which is hailed as the heart of Europe and the main channel to the Mediterranean; it maintains a stable position in the global offshore carbon emissions network. The coreness of Singapore shows a periodic tendency with the highest value of 0.245 in 2018, and it is located in Malacca Strait, which connects the Pacific and Indian oceans, being in a hub location surrounded by other core countries. As China gradually becomes the center of the global manufacturing industry, the coreness of China presents an increasing tendency, with a growth rate of 17.24% from 2015 to 2020. The major economies of the EU, such as Germany and Italy, have close carbon correlation with other core economies in the network, given their port advantages; however, some countries, such as Tonga and Guam, are weak in the shipping trade, and their coreness is almost zero.

The core positions vary for economies with different development levels, and more prosperous economies play a more pivotal role. The Group of Eight (G8); Brazil, Russia, India, China, and South Africa (BRICS); and the Asia-Pacific Economic Cooperation (APEC) are in the most central position with average coreness of 0.17, 0.13, and 0.12. G8 comprises developed countries (the United States, the United Kingdom, France, West Germany, Italy, Canada, and Japan), while BRICS are mainly emerging economies. Both organizations have frequent port trade contacts with other countries. Gross domestic product (GDP) and trade volume of the APEC organization account for over 50% of the world, including countries such as China, Singapore, the US, Japan, and Korea. Although the total coreness of the EU accounts for 9.5%, it reflects the extreme imbalance within the EU. Positions in the network of Germany, Belgium, and Spain are considerably different from those of some developing countries, such as Bulgaria and Romania. The coreness of the League of Arab States and the Association of Southeast Asian Nations (ASEAN) are the lowest since most of their member countries are in semi-edge and edge positions. Therefore, the position in the offshore carbon emissions network is mainly determined by its economic power, and countries with more advanced economies are more likely to transfer carbon to other economies.

In terms of different continents, the coreness of Asia ranks first, and the carbon correlation is larger than that of other continents. Although the number of developed countries in Asia is smaller than that of Europe, there are many big economies, such as China, Japan, India, and Korea. The geographical location is in the center of the world; thus, it is convenient for Asia to conduct its shipping trade with other countries. Europe, the continent with the most developed countries in the world, ranks second in the offshore carbon emissions network. North America has the largest economy, the US, which drives the economic development of the entire region and can affect the carbon correlation. In comparison, the continents of Africa, South America, and Oceania are in the edge positions, mainly owing to the less developed economy.

Clustering features of the offshore carbon emissions network

This section hints at the clustering features of countries in the offshore carbon emissions network, and the convergent correlations (CONCOR) algorithm in the block model could be applied to classify the countries. Four plates can be obtained from this model, and changes from 2015 to 2020 are presented in Figure 4. The typical features of all plates are that the expected internal proportion relations are smaller than the actual internal proportion relations. The received relations of plates 1, 2, and 4 are

CellPress
OPEN ACCESS

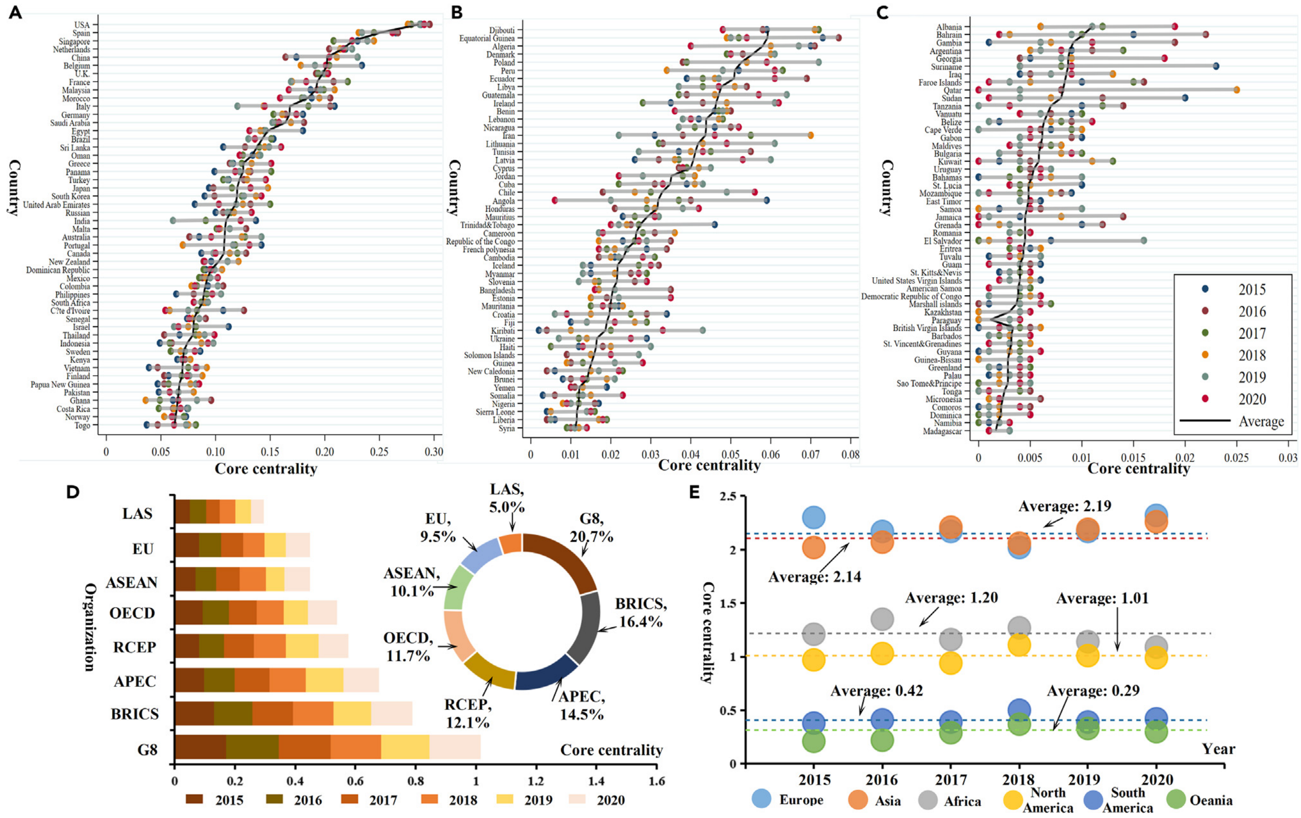

Figure 3. Coreness of countries, organizations, and continents

(A) Coreness of the top 50 countries from 2015 to 2020.

(B) Coreness of the top 51–100 countries from 2015 to 2020.

(C) Coreness of the top 101–152 countries from 2015 to 2020.

(D) Coreness of significant organizations in the world from 2015 to 2020. The bar chart represents the yearly coreness of each organization, and the pie chart represents the proportion of each organization in 2020. Coreness is calculated based on the division of total coreness of all countries by the number of countries.

(E) Coreness of different continents from 2015 to 2020; the coreness is the sum of all countries in each continent.

higher than the emitted relations, thus they belong to the main inflow plate; the received relations of plate 3 are lower than the emitted relations, thus it is the bidirectional spillover plate. Plate 1 comprises the leading economies in Asia and developed countries such as the US, Japan, Korea, Singapore, and Australia. They are located in the core positions of the network. The internal receive relations are larger than the external receive relations, and plate 1 has the lowest dependence degree on other plates. There are over 40 countries in plate 2, mainly including African countries such as Oman, the Bahamas, and Mozambique. This plate mainly receives carbon spillovers from other plates. Plate 4 contains most countries in North and South America, such as Brazil, Canada, and Chile, and it receives carbon spillovers from other plates. The main economies in the EU dominate countries in plate 3.

Based on the division of plates, cohesive subgroups analysis is performed to reveal the relationship between different plates. If the density of the plate is larger than the overall density, it indicates that this plate has a clustering tendency. Therefore, it can be assigned to 1; otherwise, it is 0. Figure 4 demonstrates that each plate is 1, and the relations with other plates are 0, implying the solid internal correlation within each plate. Although the spillovers between plates are less obvious, the offshore carbon emissions network has shown a stable state in which developed countries and large economies dominate. Less developed economies have to receive carbon spillovers from other plates.

Carbon emissions responsibility allocation

From the perspective of network structure, this decomposition problem can be transformed into how to achieve the controllability of the global offshore carbon emissions network in a more equitable, efficient, and feasible way. The carbon emissions network interacts with the emissions reduction target, and the optimal configuration can be obtained.[46] The carbon correlation calculated by the network is used to measure the relationship between different offshore carbon emissions. The technical-level and short-term needs are assumed to remain unchanged, and the external shock is the only considered factor. During the policy transmission process, with the changes in network characteristics and direct offshore carbon emissions, the carbon correlation also changes, promoting the evolution of the offshore carbon emissions network. The new network structure can, in turn, determine the total carbon emissions. According to this principle, the non-linear decomposition model is constructed to achieve the allocation of offshore carbon responsibilities. Each iteration is a time of network evolution until the

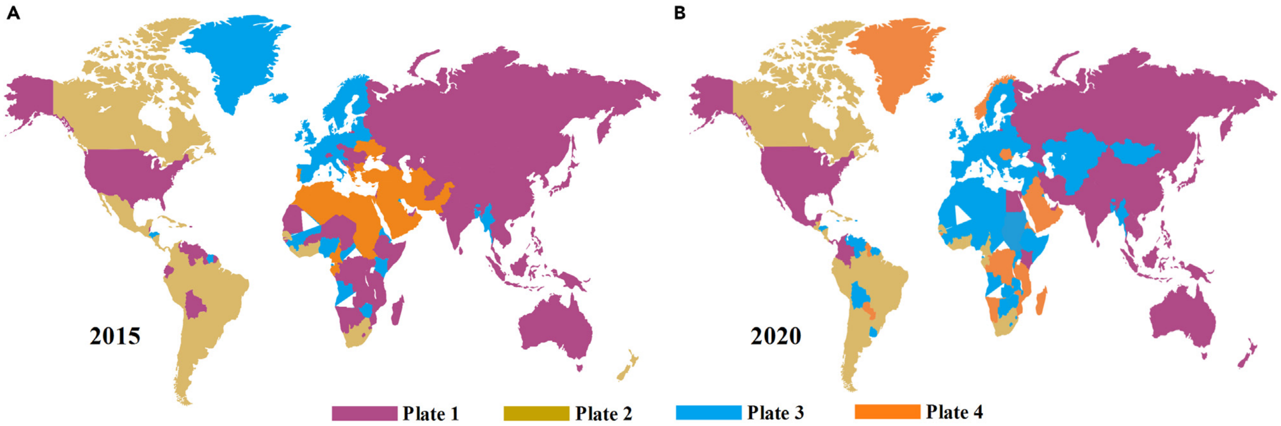

C

Year		Received relations Within	Received relations Outside	Emitted relations Within	Emitted relations Outside	Density matrix Plate 1	Density matrix Plate 2	Density matrix Plate 3	Density matrix Plate 4	Like matrix Plate 1	Like matrix Plate 2	Like matrix Plate 3	Like matrix Plate 4	
2015	Plate 1	260	210	260	184	0.17	0.02	0.03	0.02	1	0	0	0	Main inflow
	Plate 2	41	78	41	75	0.02	0.05	0	0	0	0	0	0	Main inflow
	Plate 3	298	115	298	178	0.03	0	0.13	0.03	0	0	1	0	Bidirectional spillover
	Plate 4	111	150	111	116	0.03	0	0.04	0.18	0	0	0	1	Main inflow
2016	Plate 1	228	146	228	139	0.19	0.01	0.02	0.02	1	0	0	0	Main inflow
	Plate 2	34	62	34	60	0.01	0.05	0.01	0	0	0	0	0	Main inflow
	Plate 3	335	122	335	162	0.012	0.01	0.13	0.03	0	0	1	0	Bidirectional spillover
	Plate 4	173	163	173	132	0.04	0	0.04	0.16	0	0	0	1	Main inflow
2017	Plate 1	251	199	251	173	0.16	0.02	0.03	0.02	1	0	0	0	Main inflow
	Plate 2	30	65	30	64	0.02	0.02	0	0	0	0	0	0	Main inflow
	Plate 3	259	119	259	171	0.02	0	0.14	0.03	0	0	1	0	Bidirectional spillover
	Plate 4	174	156	174	131	0.03	0	0.04	0.16	0	0	0	1	Main inflow
2018	Plate 1	276	152	276	148	0.18	0.02	0.03	0.02	1	0	0	0	Main inflow
	Plate 2	152	131	152	109	0.02	0.04	0.01	0	0	0	0	0	Main inflow
	Plate 3	198	114	198	118	0.03	0.01	0.14	0.02	0	0	1	0	Bidirectional spillover
	Plate 4	123	90	123	112	0.03	0	0.03	0.16	0	0	0	1	Main inflow
2019	Plate 1	256	181	256	175	0.13	0.02	0.03	0.01	1	0	0	0	Main inflow
	Plate 2	133	141	133	122	0.02	0.08	0.01	0.04	0	1	0	0	Main inflow
	Plate 3	135	83	135	118	0.03	0.01	0.16	0.03	0	0	1	0	Bidirectional spillover
	Plate 4	155	135	155	125	0.01	0.04	0.05	0.25	0	0	0	1	Main inflow
2020	Plate 1	236	159	236	155	0.13	0.02	0.01	0.02	1	0	0	0	Main inflow
	Plate 2	162	133	162	108	0.02	0.07	0.03	0.02	0	1	0	0	Main inflow
	Plate 3	141	98	141	142	0.02	0.03	0.18	0.05	0	0	1	0	Bidirectional spillover
	Plate 4	147	131	147	116	0.03	0.02	0.04	0.22	0	0	0	1	Main inflow

Spatial correlation: Plate 1; Plate 2; Plate 3; Plate 4. Density matrix: No density; Density. Like matrix: Density within the plate is less than the overall network density; Density within the plate is greater than the overall network density.

Figure 4. Division of different plates in the offshore carbon emissions network

(A) Map of the global offshore carbon emissions network in 2015.

(B) Map of the global offshore carbon emissions network in 2020.

(C) Spatial correlation, density matrix, and like matrix. The number of receiving relations refers to carbon emissions received by nodes in the network from other nodes, which can be divided into internal and external receiving relations of plates. The number of emitted relations refers to carbon emissions emitted by nodes in the network to other nodes, which can be divided into internal and external emitting relations of plates. The density matrix refers to the density within each plate. In the like matrix, 1 means that the density within the plate is greater than the overall network density; 0 means that the density within the plate is less than the overall network density.

goal of total quantity control is realized. The direct offshore carbon emissions are transformed into total relational quantity based on the transformation and model construction methods below. Responsibilities for carbon emission reduction can be allocated to countries using the goal of carbon emissions reduction as the external shock. This study applies the modified PSO algorithm with faster convergence speed and higher searching ability to allocate carbon emissions reduction responsibilities and ensure that the carbon emission allocation creates more output value. This approach provides a fair and reasonable mechanism for sharing responsibility for global climate governance.

The results of carbon emissions responsibility allocation are displayed in Figure 5. When the carbon emissions reduction target is set to be 5% in the next year, countries in the core positions in the network should take more responsibility for reducing offshore carbon emissions, which can make significant contributions to achieving carbon neutrality. The top five

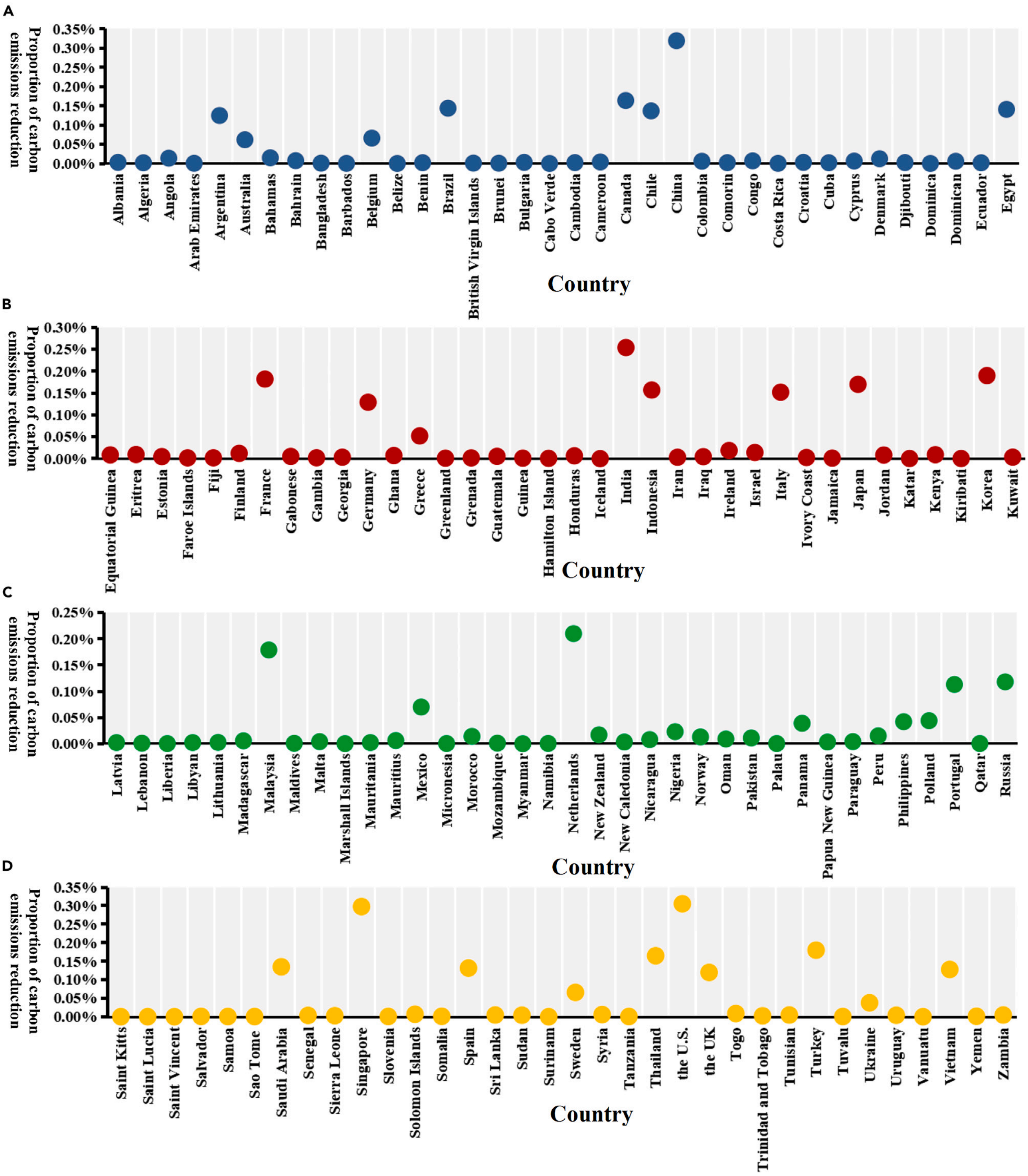

Figure 5. Allocation of carbon emissions reduction responsibility based on the offshore carbon emissions network listed by the alphabetical order of countries

(A) The proportion of carbon emissions reduction for 1–36 countries.

(B) The proportion of carbon emissions reduction for 37–72 countries.

(C) The proportion of carbon emissions reduction for 73–108 countries.

(D) The proportion of carbon emissions reduction for 109–142 countries.

countries with the largest responsibilities are China, the US, Singapore, India, and the Netherlands, with values of 0.320%, 0.305%, 0.298%, 0.254%, and 0.210%, respectively. There are 25 countries whose proportions of responsibility allocation are greater than 0.1%, and there are 95 countries whose responsibilities to reduce offshore carbon emissions are less than 0.01% owing to less advanced shipping trade, suggesting that a small group of countries dominates the global offshore carbon emissions network. Countries in more critical positions are supposed to play a leading role in accelerating the carbon emissions reduction process to create a greener world.

The above responsibilities result from the joint action of trade linkages and the spatial structure of carbon emissions with fairness and efficiency, which is similar to what Selin et al.[47] concluded. In practice, from the perspective of the current carbon emission reduction efforts of various countries, they often adopt negotiation strategies aimed at limiting their obligations while increasing the obligations of others, which significantly influences the discussion on how to solve the carbon emissions from international shipping.[48] Although it would be difficult to control all countries owing to limitations of regulatory costs, implementation assessment, and technical conditions, the allocation by relative position and relevance can balance efficiency and equity. Moreover, in terms of taking responsibility and helping to drive technological change, those top countries are more powerful than others to drive the process of reducing international carbon emissions, and they could play a political leadership role in designing and implementing the distribution of national responsibilities.[47]

DISCUSSION

Our study made several other key findings. First, the offshore carbon emissions network structure is complicated and stable, and a few countries play the leading role in the network.[9,49] In recent years, the global shipping trade is increasingly interconnected, represented by China, the EU, and the US. Accordingly, developed countries such as the US and European countries such as Germany and Italy and leading global economies, such as China and India, are in the central positions in the network and have strong network control power. Specifically, in the top 20 countries with the highest degree of centrality, there are 12 developed countries and eight emerging economies. The diffusion tendency in the offshore carbon emissions network increases and the role each country plays in the network is strengthened. Therefore, to follow the trend of globalization and improve the relationship between carbon receiving and spillovers, it is supposed to encourage international trade cooperation and make multinational policies to control carbon emissions to promote the low-carbon development of global ports. Owing to heterogeneity in the global offshore carbon emissions network, the United Nations Environment Programme and Intergovernmental Panel on Climate Change should continue their work on detecting carbon leakage and calculate the responsibility of carbon emissions reduction of each country from a network perspective.

Second, the offshore carbon emissions network shows prominent core-edge structure features, mainly determined by economic power. China, the US, Singapore, and Spain occupy the core positions. The coreness of Asia and Europe rank first and second, while Africa, South America, and Oceania are in edge positions. Countries that belong to G8, BRICS, and OPEC are in the most central positions. The above phenomenon suggests the prominent clustering features of the network, and offshore carbon emissions are mainly transferred from more developed economies to less developed economies. Our conclusions are consistent with the existing literature of Wang et al. 33[31] All countries in the network are divided into four plates with strong internal connections and weak external connections, and countries located on the same continent or with other features are on the same plate. Our conclusions suggest that countries that play the dominant role in the network may be more likely to influence other economies; differentiation policies on carbon emissions reduction should be made according to differences in factor endowment, industrial structure, and evolution trend of the network. Developed economies such as G8 are supposed to undertake more responsibilities to mitigate climate change. They also need to implement emissions reduction technologies worldwide and seek ways to substitute fossil fuels. Controlling carbon correlation from them is the key to reducing offshore carbon emissions generated from the shipping trade.

Third, based on the offshore carbon emissions network characteristics, China, the US, Singapore, India, and the Netherlands are the top five countries that should take the biggest responsibility. The purpose of allocating carbon emission reduction responsibility is to distribute a part of the total emission reduction target to each subject.[50] In the current literature, the allocation of carbon emission reduction responsibility mainly focuses on the single-equity principle,[51,52] single efficiency,[53] and combining equity and efficiency.[54] Using input-output, top-down, bottom-up, and integration methods to divide the responsibility of international carbon emissions from the perspective of production, consumption, and shared responsibility,[4,55] however, the calculations are based on the absolute values of carbon emissions instead of considering the interconnections between countries. The structural features always determine the performance of attribute data; thus, it is significant to allocate the responsibility for offshore carbon emissions reduction based on the position of each country in the network and design the global cross-regional governance mechanism. In regional collaborative reduction, the core countries in the network should be the focus to enhance information sharing and technology spillovers.

Finally, we must address the uncertainty in our data. The container liner shipping data are derived from Alphaliner Consulting, which covers 95% of global container shipping lines; however, limited by data availability, we only used 6 years of data, from 2015 to 2020. Although these data can broadly reflect the characteristics and development of global container liner shipping in recent years, more accurate and unique information might be obtainable from more time-span data and more lines. Before building the network, we performed detailed data pre-processing, including deleting the lines with incomplete information records owing to data collection errors (only a few), accurately identifying the ports and the order in the lines, etc. Moreover, we also used the country's annual container throughput statistics for network construction and made reasonable estimates for a few missing values. Although we regret not being able to obtain more microdata, such as port container throughput, the above data can better

highlight the flow and difference of maritime container trade between countries.

Based on the above conclusions, our study seeks to advance the field in several aspects. First, although previous studies focused on offshore carbon emissions,[31,56,57] they only focused on the absolute values of offshore carbon emissions and their relationships with other factors, such as human health. The extant literature did not investigate the spatial correlation of offshore carbon emissions. We address this unsolved problem by constructing a global offshore carbon emissions network based on offshore carbon emissions, trade contacts, and geographical distance. The network can visually reflect the carbon correlation along the international trade between countries since offshore carbon emissions mainly come from carbon leakage in the trade and operation of ships. Second, previous studies fail to uncover the position of each country in global offshore carbon emissions. Our study analyzes the overall and cross-regional carbon correlation and the central roles of a single country in the network. The analyses on core-edge and plate structure are conducive to identifying network characteristics and revealing its evolution tendency. Finally, allocating responsibility for carbon emissions cannot only consider countries as independent units and ignore the carbon correlation, which can cause unfairness and inaccuracy. Carbon correlation and international trade play a big part in global offshore carbon emissions, and the transferred carbon emissions should not be negligent. The existing literature uses systematic quantitative methods to study common but differentiated responsibility transfer and provide a reasonable distribution framework for developed, developing, and industrialized countries based on national and regional carbon emission accounts.[42] Equitable sharing of CO_2 emissions increases the responsibility of significant emitters, with the United States, the EU, and China bearing much higher Chinese Depositary Receipt (CDR) liabilities than other countries.[58] Based on the constructed offshore carbon emissions network and intelligent optimization algorithm, this paper recalculates the offshore carbon emissions allocation responsibility with 5% carbon emission reduction as the target.

Limitations of the study

Despite the contributions of this study, it inevitably has some limitations and unaddressed issues. First, although our basic data cover 95% of global container shipping lines, the calculated results represent carbon transfer between ports instead of carbon emissions of the port itself. In other words, when calculating port carbon emissions of each country, we only consider carbon emissions from shipping, which cannot capture all carbon emissions of the port itself in a micro-perspective. Port carbon emissions should be expanded to a wider range that also include carbon emissions directly from ports, although it just accounts for a very small part of the total port carbon emissions. Second, due to data limitation, we did not explore the driving factors of port carbon emissions network evolution, and we calculate the total port carbon emissions of a country. In fact, the research objects can be expanded to each port to evaluate their roles in the network. Finally, our focus is on the global scale, and what is interesting as well is to study the port carbon emissions of particular economies, organizations, or regions, which can draw more meaningful conclusions to guide the regional collaborative reduction of carbon emissions.

EXPERIMENTAL PROCEDURES

Resource availability

Materials availability

This study did not generate new unique reagents.

Data

This study applies the records of about 1,700 container shipping lines, covering 98% of global container shipping lines during 2015 and 2020, to estimate the carbon emissions of each line and calculate the total offshore carbon emissions of each country. Data on global container shipping lines come from the website of Alphaliner[61] based on the commercial license. For each service line, a list of ports a container ship consecutively calls during the service is provided. The service line data also include service duration, frequency, traffic capacity, ship types, etc. Carbon emissions generated at the port in each line are calculated according to different states of ships, and data related to the states of ships and carbon emissions factors can be obtained from the IMO fourth GHG Report.[3] The container throughput and geographical distance data are used to construct the offshore carbon emissions network. Data on container throughput during 2015 and 2020 of all countries are from the World Bank.[62] The geographical distance data between the countries are obtained from the Centre D'études Prospectives et D'informations Internationals (CEPII) Databases.[63]

Method

Calculation of offshore carbon emissions. According to the speed of ships and distance from the coastline, the navigation process of container transportation mainly includes three parts: cruising, maneuvering, and berthing. For the entire transportation process near the coast, ports are mainly faced with the latter two states of ships, the two sources of offshore carbon emissions. We assume that each container ship has one propulsion engine, auxiliary engine, and boiler and that only auxiliary engines and boilers operate when container ships are berthing in ports. All three engines work during maneuvering modes.[3] Thus, carbon emissions of voyage w in the yth year $E_{w,y}$ are calculated by Equation 1 [2,57,64]:

$$E_{w,y} = \left[T_{man,w} \times (ME_{install,w} \times LF_{man,w} \times \varepsilon_p + AE_{man,w} + BO_{man,w}) + T_{ber} \times (AE_{ber,w} + BO_{ber,w})\right] \times AC_w \times EF_{co_2} \quad \text{(Equation 1)}$$

where $ME_{install,w}$ denotes the average design power of the propulsion engine on voyage w, which changes with container ships' carrying capacity. $LF_{man,w}$ represents loading factors of the propulsion engine on voyage w during maneuvering; loading factor is the ratio between the engine's output power and its maximum continuous rating (MCR) (from Ship Business[65]). $LF_{man,w} = (V_{man,w}/V_{design,w})^3$, where $V_{man,w}$ is the average speed of the container ship during maneuvering and $V_{design,w}$ is the maximum design speed of the container ship, which is determined by the standard ship's maximum carrying capacity on each route; we define the representative ship type as the standard ship on each line because different types of ship have sailed in a different line. The values of $ME_{install,w}$, $V_{man,w}$, and $V_{design,w}$ utilized in this paper are derived from the fourth IMO study on GHG emissions (Table S2). ε_p is the ratio of MCR to the install power of the propulsion engine. Based on the fourth IMO GHG report, the most fuel-efficient point for a slow-speed diesel built after 2001 was 175 g/kWh.[3] The unique specific fuel consumption value was 195 g/kWh[3] for any energy-based emission factors; thus, the reducing factor is 0.90 (=175/195). $AE_{man,w}$ and $AE_{ber,w}$ represent the power output of auxiliary engines during maneuvering and berthing on voyage w. $BO_{man,w}$ and $BO_{ber,w}$ represent the power output of boilers during maneuvering and berthing on voyage w. AC_w is the annual cycle of voyage w, and $AC_w = |365/F_w|$, where F_w represents the departure interval of each container ship on voyage w from the departure port. EF_{co2} is the emission factor of CO_2.[3] $T_{man,w}$ and $T_{ber,w}$ represent the active time of container ships during maneuvering and berthing. Based on the IMO report, the time corresponding to berthing and departure stages of maneuvering in port is generally less than 1 h; thus, we assumed that, for container ships, every port call is fixed to 1 h. 33[31] $T_{man,w}$ for all voyages is set to be 1, and $T_{ber,w}$ is calculated by Equation 2:

$$T_{ber,w} = T_w - T_{man,w} - T_{cru,w} \quad \text{(Equation 2)}$$

where T_w is the total duration of the voyage w. $T_{cru,w}$ is the duration of cruising on voyage w, and $T_{cru,w} = D_w / V_{cur,w}$, where D_w is the distance of voyage w. $V_{cur,w}$ is the average speed of the container ship during cruising on voyage w, and its value denotes container ships with different loads (Table S2).

Offshore carbon emissions from each route are evenly distributed to each port. Carbon emissions $E_{p,w}$ of port p of voyage w are calculated by Equation 3, and the total offshore carbon emissions of a country in the yth year are calculated by Equation 4:

$$E_{p,w} = \frac{E_{w,y}}{m_w} \quad \text{(Equation 3)}$$

$$EP_{p,y} = \sum_{w} E_{p,w} \quad \text{(Equation 4)}$$

Construction of offshore carbon emissions network. Among ports in the global trade routes, countries, including ports, are denoted as the network nodes, and the offshore carbon emissions flow embedded in the global trade between countries are edges of the network.[66] Notably, the network represents the spatial association of offshore emissions that belong to countries (ports) owing to international container shipping trade, which could implement responsibility allocation and policies more effectively by having clear countries and emissions. Global offshore carbon emissions system of ports can be represented as:

$$G = (V, E) \quad \text{(Equation 5)}$$

where $V = \{v_1, v_2, \cdots, v_n\}$ is the node set of the network, and n indicates the total number of nodes; v_n represents the countries to which the ports belong. $E = \{e_{ij}\}_{n\times n}$ is the edge set of the network, indicating the carbon emissions flow relationship between countries. Each edge e_{ij} in E has a pair of nodes in (v_i, v_j) from V.

To better investigate the spatial-temporal evolution trend and complex network characteristics of global offshore carbon emissions, this study constructs the offshore carbon emissions network from 2015 to 2020 by applying the modified gravity model to describe the interactive relationship between countries and uncover the dynamic evolution characteristics of the network structure.[15] Generally, the gravity model can integrate various factors, such as geographical distance, economic development level, and population size, to reflect the relationship strength of two regions. According to different application scenarios and research problems, it can be modified and reconstructed to include the most relevant factors. This study describes the structural characteristics of the offshore carbon emission network in the process of global liner shipping by constructing the offshore carbon emissions gravity model (PCEG). The PCEG reflects the spatial correlation of carbon emissions between countries; offshore carbon emissions were included by expanding the original form. Based on relevant studies,[67,68] we revise the gravity model using calculated offshore carbon emissions from ports. The container throughput always represents the output level of a port 66[69]; thus, it is used to represent the economic development of a port. Furthermore, the study subject is national offshore emissions; the number of ports in a country broadly represents the shipping scale,[67] which is also an essential factor affecting the emissions, because the development of shipping in various countries is significantly unbalanced. Therefore, the PCEG model includes variables of offshore carbon emissions, the number of ports, and the container throughput of each port owing to their importance in affecting the carbon emissions of ports. Considering the vast differences and asymmetry of the frequency of trade contacts between countries, the empirical constant in the gravity model is corrected using the ratio of the national shipping frequency to the total global shipping frequency. The factor that measures the trade contacts is introduced in the gravity model to highlight the status of countries with trade contacts. The equation of the PCEG model is Equation 8:

$$x_{ij} = \frac{K_i}{(K_i + K_j)} \times \frac{\sqrt{E_i T_i N_i} \times \sqrt{E_j T_j N_j}}{D_{ij}^2} \times C_{ij} \quad \text{(Equation 8)}$$

where x_{ij} represents the correlation between countries i and j. K_i and K_j are the trade frequency of ports of country i and j, represented by the number of passing routes, and $K_i/(K_i + K_j)$ is the correction factor of the PCEG model. E_i and E_j are the offshore carbon emissions of countries I and j. T_i and T_j are the port container throughput of countries i and j; N_i and N_j are the numbers of ports of countries i and j. D_{ij} is the distance between countries i and j. C_{ij} is whether there are trade contacts between countries i and j, and, if the route connects the two countries, its value is 1; otherwise, it is 0.

Calculation of network characteristics. The network analysis can identify the influential actors in the whole network and analyze the network structure.[66,70] This study applies the network analysis to reveal the overall network characteristics, the role of individual countries in the network, and the spatial features. Indicators that can reflect the features of the overall network and individual countries are summarized in Table S3. Network correlation, network hierarchy, and network efficiency are used to measure the overall network characteristics, and degree and betweenness centrality are applied to reflect the individual features of each country. The core-edge analysis can reveal the network structure by identifying the influence of crucial nodes.[71,72] In the network, many nodes can become the core nodes affected by various factors, while other nodes will gradually move to the edge owing to slow development speed. The development of two types of nodes is imbalanced in that core nodes play a dominant role in the network, and edge nodes depend greatly on core nodes. This study employs the core-edge analysis to uncover whether the offshore carbon emissions network shows clear core-edge structure features.

The block model can be used to uncover the spatial features,[73,74] and it divides the network into four types: bidirectional spillover plate, main inflow plate, main outflow plate, and broker plate, according to the number of receiving and sending relations inside and outside the plate and the number of members inside the plate, as shown in Table S4. A CONCOR method is applied to determine the maximum segment depth and the convergence standard,[75] which is separately 2 and 0.2.

Carbon emissions responsibility allocation method. Based on the constructed offshore carbon emissions network, how to allocate the offshore carbon emissions responsibility to each country will be investigated. The flow of offshore carbon emissions between countries results from various complex factors, and the decomposition of carbon emissions responsibility based on the relational data considers the co-movement relation of countries. The decomposition model[76] to represent the relationship between total relational quantity and actual direct offshore carbon emissions is:

$$DCI'_n = DCI_n - \sum \frac{DCE_i x_i}{q_i} \sum_{m=1}^{l} \prod_{(j,k) \in path_n(m)} w[j,k] \quad \text{(Equation 9)}$$

$$TCE^t = \begin{bmatrix} DCI_1 & \dots & 0 \\ \dots & \dots & \dots \\ 0 & \dots & DCI_n \end{bmatrix} \times L^d \begin{bmatrix} Y_1^d & \dots & 0 \\ \dots & \dots & \dots \\ 0 & \dots & Y_n^d \end{bmatrix} \quad \text{(Equation 10)}$$

$$F(X) = E - Sum\left(TCE^t\right) \quad \text{(Equation 11)}$$

where i is a controlled country and n is other countries. DCI_n is the direct offshore carbon emissions intensity, and DCI'_n is the direct offshore carbon emissions intensity after being attacked by policies. DCE_i is the direct offshore carbon emissions, x_i is the target of carbon emissions reduction, and q_i is the GDP of each country. l is the number of paths from a controlled country to other countries. $path_n(m)$ is the mth path from country r to n. $w[j,k]$ is the weight of two paths, representing the non-linear impact between countries. E is the total relational quantity obtained from the offshore carbon emissions network. $Sum(TCE^t)$ is the weight of the carbon emissions network, and $F(X)$ is the gap between the sum of weights and the total relational quantity.

The non-linear algorithm of PSO is applied to achieve the offshore carbon emissions responsibility allocation, which has a fast search speed and high efficiency.[77,78] Specific steps are as follows. First, the carbon correlation matrix obtained above is normalized into a 0–1 matrix. Second, initialize the PSO algorithm by ranking offshore carbon emissions of all countries in descending order, including the population position and flying velocity of particles as shown in Equations 12 and 13:

$$x_{id}^t = [x_{i1}, x_{i2}, x_{i3}, x_{i4}, x_{i5}, \cdots, x_{id}]^t, i = 1,2,3,\cdots,I, t = 1,2,3,\cdots,T, d = D \quad \text{(Equation 12)}$$

$$v_{id}^t = [v_{i1}, v_{i2}, v_{i3}, v_{i4}, v_{i5}, \cdots, v_{id}]^t, i = 1,2,3,\cdots,I, t = 1,2,3,\cdots,T, d = D \quad \text{(Equation 13)}$$

where I is the number of particles, T is the number of iterations, and D is the dimension of the vector.

Third, calculate the cost function of each particle in the tth iteration according to Equation 14.

$$F(X) = E - Sum(TCE) \quad \text{(Equation 14)}$$

where E is the value after the total amount of offshore carbon emissions correlation decreases by 5% in 2021; *Sum (TCE)* is the weighted sum of the carbon emissions network. In the cost function *F(X)*, X is the national target matrix of emissions reduction. When the cost function value approximates 0, it is closer to the optimal emissions reduction target of each country.

Fourth, update the position and speed of each particle. Considering that the update mode of position and speed of the original PSO algorithm can result in a low convergence rate, the inertia weight parameter is introduced to adjust the particle velocity flexibly, and the update formula is:

$$v_{id}^{t+1} = wv_{id}^{t} + c_1 r_1 \left(pbest_{id}^{t} - x_{id}^{t}\right) + c_2 r_2 \left(gbest_{d}^{t} - x_{id}^{t}\right) \quad \text{(Equation 15)}$$

where w is the inertial weight parameter. t is the current iteration number, and T is the maximum number of iterations. w_{max} and w_{min} are the maximum and minimum inertia weight, and, according to the experiment, their value is separately set to be 0.9 and 0.4.[77] c_1 and c_2 are the learning factor, r_1 and r_2 are the random number in [0,1],[79] *pbest* is the local optimum, and *gbest* is the global best position of the particle.

Finally, judge whether the PSO algorithm meets the termination condition. If the iteration number exceeds T or finds the optimal solution of the cost function, the algorithm ends; otherwise, the velocity and position are updated to find the optimal solution. After iterations of the network, the allocation of carbon emissions responsibility can be determined.

Data and code availability

All the data used and measured are available on the figshare (https://figshare.com/) scientific repository.[59] The network was constructed and analyzed using Gephi0.9.2.[60]

SUPPLEMENTAL INFORMATION

Supplemental information can be found online at https://doi.org/10.1016/j.patter.2023.100801.

ACKNOWLEDGMENTS

This research was funded by the National Natural Science Foundation of China (grant number 72073018; 72261147705; 72022019; T2241025), the Major Program of National Fund of Philosophy and Social Science Foundation of China (grant number 20&ZD129), and Liaoning Revitalization Talents Program of China (grant number XLYC2007191).

AUTHOR CONTRIBUTIONS

B.L., conceptualization, methodology, funding acquisition, supervision; Y.S., data curation, visualization, software, methodology, formal analysis, writing – review & editing; L.F., visualization, software, methodology, formal analysis, writing – original draft; X.M., conceptualization, methodology, result analysis, writing – review. H.D., conceptualization and review.

DECLARATION OF INTERESTS

The authors declare no competing interests.

Received: December 12, 2022
Revised: April 3, 2023
Accepted: June 27, 2023
Published: July 31, 2023

REFERENCES

1. United Nations Conference on Trade and Development (2019). Review of Maritime Transport 2017.
2. Wang, X.T., Liu, H., Lv, Z.F., Deng, F.Y., Xu, H.L., Qi, L.J., Shi, M.S., Zhao, J.C., Zheng, S.X., Man, H.Y., and He, K.B. (2021). Trade-linked shipping CO2 emissions. Nat. Clim. Change *11*, 945–951. https://doi.org/10.1038/s41558-021-01176-6.
3. International Maritime Organization (2020). Fourth IMO Greenhouse Gas Study.
4. Liu, H., Meng, Z.H., Lv, Z.F., Wang, X.T., Deng, F.Y., Liu, Y., Zhang, Y.N., Shi, M.S., Zhang, Q., and He, K.B. (2019). Emissions and health impacts from global shipping embodied in US–China bilateral trade. Nat. Sustain. *2*, 1027–1033. https://doi.org/10.1038/s41893-019-0414-z.
5. Bouman, E.A., Lindstad, E., Rialland, A.I., and Strømman, A.H. (2017). State-of-the-art technologies, measures, and potential for reducing GHG emissions from shipping – A review. Transport. Res. Transport Environ. *52*, 408–421. https://doi.org/10.1016/j.trd.2017.03.022.
6. Hoang, A.T., Foley, A.M., Nižetić, S., Huang, Z., Ong, H.C., Ölçer, A.I., Pham, V.V., and Nguyen, X.P. (2022). Energy-related approach for reduction of CO2 emissions: A critical strategy on the port-to-ship pathway. J. Clean. Prod. *355*, 131772. https://doi.org/10.1016/j.jclepro.2022.131772.
7. Hassanpouryouzband, A., Joonaki, E., Edlmann, K., and Haszeldine, R.S. (2021). Offshore Geological Storage of Hydrogen: Is This Our Best Option to Achieve Net-Zero? ACS Energy Lett. *6*, 2181–2186. https://doi.org/10.1021/acsenergylett.1c00845.
8. Liu, H., Yang, X., Cheng, X., Zhao, G., Zheng, G., Li, X., and Bräunl, T. (2021). A net-zero future for freight. One Earth *12*, 1517–1519. https://doi.org/10.1016/j.oneear.2021.11.001.
9. Li, Y.L., Chen, B., and Chen, G.Q. (2020). Carbon network embodied in international trade: Global structural evolution and its policy implications. Energy Pol. *139*, 111316. https://doi.org/10.1016/j.enpol.2020.111316.
10. Tang, R., Zhao, J., Liu, Y., Huang, X., Zhang, Y., Zhou, D., Ding, A., Nielsen, C.P., and Wang, H. (2022). Air quality and health co-benefits of China's carbon dioxide emissions peaking before 2030. Nat. Commun. *13*, 1008–1009. https://doi.org/10.1038/s41467-022-28672-3.
11. Wu, M., Li, K.X., Xiao, Y., and Yuen, K.F. (2022). Carbon Emission Trading Scheme in the shipping sector: Drivers, challenges, and impacts. Mar. Pol. *138*, 104989. https://doi.org/10.1016/j.marpol.2022.104989.
12. Radmehr, R., Henneberry, S.R., and Shayanmehr, S. (2021). Renewable Energy Consumption, CO2 Emissions, and Economic Growth Nexus: A Simultaneity Spatial Modeling Analysis of EU Countries. Struct. Change Econ. Dynam. *57*, 13–27. https://doi.org/10.1016/j.strueco.2021.01.006.
13. You, W., and Lv, Z. (2018). Spillover effects of economic globalization on CO2 emissions: A spatial panel approach. Energy Econ. *73*, 248–257. https://doi.org/10.1016/j.eneco.2018.05.016.
14. Zhong, Z., Jiang, L., and Zhou, P. (2018). Transnational transfer of carbon emissions embodied in trade: Characteristics and determinants from a spatial perspective. Energy *147*, 858–875. https://doi.org/10.1016/j.energy.2018.01.008.
15. Liu, S., and Xiao, Q. (2021). An empirical analysis on spatial correlation investigation of industrial carbon emissions using SNA-ICE model. Energy *224*, 120183. https://doi.org/10.1016/j.energy.2021.120183.
16. Tang, L., Wu, J., Yu, L., and Bao, Q. (2017). Carbon allowance auction design of China's emissions trading scheme: A multi-agent-based approach. Energy Pol. *102*, 30–40. https://doi.org/10.1016/j.enpol.2016.11.041.
17. Yi, W.J., Zou, L.L., Wei, Y.M.L., Guo, J., Wang, K., and Wei, Y.M. (2011). How can China reach its CO2 intensity reduction targets by 2020? A regional allocation based on equity and development. Energy Pol. *39*, 2407–2415. https://doi.org/10.1016/j.enpol.2011.01.063.
18. Chen, W., and Lin, H. (2016). Intersectoral burden sharing of CO2 mitigation in China in 2020. Noncoding. RNA *3*, 1–14. https://doi.org/10.1007/s11027-014-9566-3.
19. Sasse, J.P., and Trutnevyte, E. (2019). Distributional trade-offs between regionally equitable and cost-efficient allocation of renewable electricity

generation. Appl. Energy *254*, 113724. https://doi.org/10.1016/j.apenergy.2019.113724.

20. Feng, C., Chu, F., Ding, J., Bi, G., and Liang, L. (2015). Carbon Emissions Abatement (CEA) allocation and compensation schemes based on DEA. Omega (United Kingdom) *53*, 78–89. https://doi.org/10.1016/j.omega.2014.12.005.
21. Zhang, Y., Bao, Y.L., Wu, Y., Yu, C.L., Huang, Y.X., Sun, Y., Zheng, L.H., and Li, Y.X. (2013). The responsibility for carbon emissions and carbon efficiency at the sectoral level: Evidence from China. Mol. Med. Rep. *8*, 967–972. https://doi.org/10.1016/j.eneco.2013.05.025.
22. Li, Y., Wei, Y., Zhang, X., and Tao, Y. (2020). Regional and provincial CO2 emission reduction task decomposition of China's 2030 carbon emission peak based on the efficiency, equity and synthesizing principles. Struct. Change Econ. Dynam. *53*, 237–256. https://doi.org/10.1016/j.strueco.2020.02.007.
23. Hu, Y. (2020). Empirical Study on Industrial Decomposition Method of Carbon Emissions Cap Control Target Based on Complex Network Perspective. https://doi.org/10.1016/j.techfore.2021.121030.
24. United Nations Climate Change (1998). Kyoto Protocol.
25. den Elzen, M.G.J., Beltran, A.M., Hof, A.F., van Ruijven, B., and van Vliet, J. (2013). Reduction targets and abatement costs of developing countries resulting from global and developed countries' reduction targets by 2050. Mitig. Adapt. Strategies Glob. Change *18*, 491–512. https://doi.org/10.1007/s11027-012-9371-9.
26. Mi, Z., Zheng, J., Green, F., Guan, D., Meng, J., Feng, K., Liang, X., and Wang, S. (2021). Decoupling without outsourcing? How China's consumption-based CO2 emissions have plateaued. iScience *24*, 103130. https://doi.org/10.1016/j.isci.2021.103130.
27. United Nations Conference on Trade and Development (2020). Review of Maritime Transport 2020.
28. Liao, C.H., Tseng, P.H., Cullinane, K., and Lu, C.S. (2010). The impact of an emerging port on the carbon dioxide emissions of inland container transport: An empirical study of Taipei port. Energy Pol. *38*, 5251–5257. https://doi.org/10.1016/j.enpol.2010.05.018.
29. Liu, H., Meng, Z.H., Shang, Y., Lv, Z.F., Jin, X.X., Fu, M.L., and He, K.B. (2018). Shipping emission forecasts and cost-benefit analysis of China ports and key regions' control. Environ. Pollut. *236*, 49–59. https://doi.org/10.1016/j.envpol.2018.01.018.
30. Freitag, C., Berners-Lee, M., Widdicks, K., Knowles, B., Blair, G.S., and Friday, A. (2021). The real climate and transformative impact of ICT: A critique of estimates, trends, and regulations. Patterns *2*, 100340. https://doi.org/10.1016/j.patter.2021.100340.
31. Wang, L., Peng, C., Shi, W., and Zhu, M. (2020). Carbon dioxide emissions from port container distribution: Spatial characteristics and driving factors. Transport. Res. Transport Environ. *82*, 102318. https://doi.org/10.1016/j.trd.2020.102318.
32. Wang, Z., Su, B., Xie, R., and Long, H. (2020). China's aggregate embodied CO2 emission intensity from 2007 to 2012: A multi-region multiplicative structural decomposition analysis. Energy Econ. *85*, 104568. https://doi.org/10.1016/j.eneco.2019.104568.
33. Wu, K., Yang, T., and Wei, X. (2019). Does the structure of intersectoral embedded carbon flow network matter to carbon reduction in China? J. Clean. Prod. *233*, 1061–1074. https://doi.org/10.1016/j.jclepro.2019.05.091.
34. Pan, X., Teng, F., Ha, Y., and Wang, G. (2014). Equitable Access to Sustainable Development: Based on the comparative study of carbon emission rights allocation schemes. Appl. Energy *130*, 632–640. https://doi.org/10.1016/j.apenergy.2014.03.072.
35. Wang, K., Zhang, X., Wei, Y.M., and Yu, S. (2013). Regional allocation of CO2 emissions allowance over provinces in China by 2020. Energy Pol. *54*, 214–229. https://doi.org/10.1016/j.enpol.2012.11.030.
36. Xie, Q., Hu, P., Jiang, A., and Li, Y. (2019). Carbon emissions allocation based on satisfaction perspective and data envelopment analysis. Energy Pol. *132*, 254–264. https://doi.org/10.1016/j.enpol.2019.05.024.
37. Sun, J., Karmazyn, B., Lin, J., Faught, P.R., Fan, R., and Saxena, R. (2014). Allocation of emission permits using DEA: Centralised and individual points of view. J. Pediatr. *164*, 419–420. https://doi.org/10.1080/00207543.2013.829592.
38. Li, Y., Wang, Y.z., and Cui, Q. (2015). Evaluating airline efficiency: An application of Virtual Frontier Network SBM. Transport. Res. Part E Logist. Transp. Rev. *81*, 1–17. https://doi.org/10.1016/j.tre.2015.06.006.
39. Zhou, Y., Zhang, J., Zhou, H., Hu, X., Zhang, L., and Zhang, M. (2013). Environmental efficiency analysis of power industry in China based on an entropy SBM model. Energy Pol. *106*, 68–72. https://doi.org/10.1016/j.enpol.2012.09.060.
40. Wang, R., Wang, Q., and Yao, S. (2021). Evaluation and difference analysis of regional energy efficiency in China under the carbon neutrality targets: Insights from DEA and Theil models. J. Environ. Manag. *293*, 112958. https://doi.org/10.1016/j.jenvman.2021.112958.
41. Gopalakrishnan, S., Granot, D., Granot, F., Sošić, G., and Cui, H. (2021). Incentives and emission responsibility allocation in supply chains. Manag. Sci. *67*, 4172–4190. https://doi.org/10.1287/mnsc.2020.3724.
42. Wei, Y.M., Wang, L., Liao, H., Wang, K., Murty, T., and Yan, J. (2014). Responsibility accounting in carbon allocation: A global perspective. Appl. Energy *130*, 122–133. https://doi.org/10.1016/j.apenergy.2014.05.025.
43. Yu, Z., Geng, Y., Dai, H., Wu, R., Liu, Z., Tian, X., and Bleischwitz, R. (2018). A general equilibrium analysis on the impacts of regional and sectoral emission allowance allocation at carbon trading market. J. Clean. Prod. *192*, 421–432. https://doi.org/10.1016/j.jclepro.2018.05.006.
44. Tao, J., and Kong, X. (2021). Spatial allocation of anthropogenic carbon dioxide emission statistics data fusing multi-source data based on Bayesian network. Sci. Rep. *11*, 18128–18216. https://doi.org/10.1038/s41598-021-93456-6.
45. Liu, Y., Wei, X., Chen, W., Hu, L., and He, Z. (2021). A graph-traversal approach to identify influential nodes in a network. Patterns *2*, 100321. https://doi.org/10.1016/j.patter.2021.100321.
46. Feng, Z.k., Niu, W.j., and Cheng, C.t. (2017). Multi-objective quantum-behaved particle swarm optimization for economic environmental hydrothermal energy system scheduling. Energy *131*, 165–178. https://doi.org/10.1016/j.energy.2017.05.013.
47. Selin, H., Zhang, Y., Dunn, R., Selin, N.E., and Lau, A.K.H. (2021). Mitigation of CO2emissions from international shipping through national allocation. Environ. Res. Lett. *16*, 045009. https://doi.org/10.1088/1748-9326/abec02.
48. Bows-Larkin, A. (2015). All adrift: aviation, shipping, and climate change policy. Clim. Pol. *15*, 681–702. https://doi.org/10.1080/14693062.2014.965125.
49. Ma, R., Li, K., Guo, Y., Zhang, B., Zhao, X., Linder, S., Guan, C., Chen, G., Gan, Y., and Meng, J. (2021). Mitigation potential of global ammonia emissions and related health impacts in the trade network. Nat. Commun. *12*, 6308–6313. https://doi.org/10.1038/s41467-021-25854-3.
50. Qin, Q., Liu, Y., and Huang, J.P. (2020). A cooperative game analysis for the allocation of carbon emissions reduction responsibility in China's power industry. Energy Econ. *92*, 104960. https://doi.org/10.1016/j.eneco.2020.104960.
51. Zetterberg, L., Wråke, M., Sterner, T., Fischer, C., and Burtraw, D. (2012). Short-run allocation of emissions allowances and long-term goals for climate policy. Ambio *41*, 23–32. https://doi.org/10.1007/s13280-011-0238-1.
52. Schmidt, R.C., and Heitzig, J. (2014). Carbon leakage: Grandfathering as an incentive device to avert firm relocation. J. Environ. Econ. Manag. *67*, 209–223. https://doi.org/10.1016/j.jeem.2013.12.004.
53. Fang, K., Zhang, Q., Long, Y., Yoshida, Y., Sun, L., Zhang, H., Dou, Y., and Li, S. (2019). How can China achieve its Intended Nationally Determined Contributions by 2030? A multi-criteria allocation of China's carbon emission allowance. Appl. Energy *241*, 380–389. https://doi.org/10.1016/j.apenergy.2019.03.055.

CellPress
OPEN ACCESS

54. Zhan, D. (2022). Allocation of carbon emission quotas among provinces in China: efficiency, fairness and balanced allocation. Environ. Sci. Pollut. Res. Int. *29*, 21692–21704. https://doi.org/10.1007/s11356-021-17309-5.

55. Wu, J., Guo, Q., Yuan, J., Lin, J., Xiao, L., and Yang, D. (2019). An integrated approach for allocating carbon emission quotas in China's emissions trading system. Resour. Conserv. Recycl. *143*, 291–298. https://doi.org/10.1016/j.resconrec.2019.01.001.

56. Liu, H., Fu, M., Jin, X., Shang, Y., Shindell, D., Faluvegi, G., Shindell, C., and He, K. (2016). Health and climate impacts of ocean-going vessels in East Asia. Nat. Clim. Change *6*, 1037–1041. https://doi.org/10.1038/nclimate3083.

57. Zhang, Y., Eastham, S.D., Lau, A.K., Fung, J.C., and Selin, N.E. (2021). Global air quality and health impacts of domestic and international shipping. Environ. Res. Lett. *16*, 084055. https://doi.org/10.1088/1748-9326/ac146b.

58. Fyson, C.L., Baur, S., Gidden, M., and Schleussner, C.F. (2020). Fair-share carbon dioxide removal increases major emitter responsibility. Nat. Clim. Change *10*, 836–841. https://doi.org/10.1038/s41558-020-0857-2.

59. Lu, B., Sun, Y., Fan, L., Ma, X., and Duan, H. (2023). https://doi.org/10.6084/m9.figshare.22999841.v1.

60. Mathieu, B., Sebastien, H., and Mathieu, J. (2009). Gephi: an open source software for exploring and manipulating networks. In International AAAI Conference on Weblogs and Social Media.

61. Alphaliner http://www.alphaliner.com/.

62. Bank, T.W. https://data.worldbank.org/indicator/IS.SHP.GOOD.TU?end=2020&start=2000.

63. CEPII http://www.cepii.fr/CEPII/en/bdd_modele/bdd_modele_item.asp?id=6.

64. Kramel, D., Muri, H., Kim, Y., Lonka, R., Nielsen, J.B., Ringvold, A.L., Bouman, E.A., Steen, S., and Strømman, A.H. (2021). Global Shipping Emissions from a Well-to-Wake Perspective: The MariTEAM Model. Environ. Sci. Technol. *55*, 15040–15050. https://doi.org/10.1021/acs.est.1c03937.

65. Business, S. http://shipsbusiness.com/engine-load-management.html.

66. Scott, J. (2007). Social Network Analysis: A Handbook (Sage Publication).

67. He, Y.Y., Wei, Z.X., Liu, G.Q., and Zhou, P. (2020). Spatial network analysis of carbon emissions from the electricity sector in China. J. Clean. Prod. *262*, 121193. https://doi.org/10.1016/j.jclepro.2020.121193.

68. Jiang, Q., and Ma, X. (2021). Spillovers of environmental regulation on carbon emissions network. Technol. Forecast. Soc. Change *169*, 120825. https://doi.org/10.1016/j.techfore.2021.120825.

69. Chang, Y.T., Jo, A., Choi, K.S., and Lee, S. (2021). Port efficiency and international trade in China. Transp. A Transp. Sci. *17*, 801–823. https://doi.org/10.1080/23249935.2020.1817169.

70. Yustiawan, Y., Maharani, W., and Gozali, A.A. (2015). Degree Centrality for Social Network with Opsahl Method. Procedia Comput. Sci. *59*, 419–426. https://doi.org/10.1016/j.procs.2015.07.559.

71. Boguñá, M., Pastor-Satorras, R., Díaz-Guilera, A., and Arenas, A. (2004). Models of social networks based on social distance attachment. Phys. Rev. E *70*, 8. https://doi.org/10.1103/PhysRevE.70.056122.

72. Friedmann, J. (1966). Regional Development Policy: A Case Study of Venezuela (MIT Press).

73. White, H.C., Boorman, S.A., and Breiger, R.L. (1976). Social structure from multiple networks. I. Blockmodels of roles and positions. Am. J. Sociol. *81*, 730–780. https://doi.org/10.1086/226141.

74. Smith, D.A., and White, D.R. (1992). Structure and dynamics of the global economy: network analysis of international trade. Soc. Forces *70*, 857–893.

75. Sun, L., Qin, L., Taghizadeh-Hesary, F., Zhang, J., Mohsin, M., and Chaudhry, I.S. (2020). Analyzing carbon emission transfer network structure among provinces in China: new evidence from social network analysis. Environ. Sci. Pollut. Res. Int. *27*, 23281–23300. https://doi.org/10.1007/s11356-020-08911-0.

76. Zhang, M., Mu, H., Ning, Y., and Song, Y. (2009). Decomposition of energy-related CO2 emission over 1991-2006 in China. Ecol. Econ. *68*, 2122–2128. https://doi.org/10.1016/j.ecolecon.2009.02.005.

77. Van Den Bergh, F., and Engelbrecht, A. (2006). A study of particle swarm optimization particle trajectories. Inf. Sci. *176*, 937–971. https://doi.org/10.1016/j.ins.2005.02.003.

78. Shi, Y., and Eberhart, R. (2009). Monitoring of particle swarm optimization. Front. Comput. Sci. China *3*, 31–37. https://doi.org/10.1007/s11704-009-0008-4.

79. Ma, Y., Zhong, P.a., Xu, B., Zhu, F., Lu, Q., and Wang, H. (2021). Spark-based parallel dynamic programming and particle swarm optimization via cloud computing for a large-scale reservoir system. J. Hydrol. X. *598*, 126444. https://doi.org/10.1016/j.jhydrol.2021.126444.

Patterns, Volume *4*

Supplemental information

Evolutionary characteristics of global offshore carbon emissions network and responsibility allocation of emissions reduction

Bo Lu, Yue Sun, Lijie Fan, Xuejiao Ma, and Hongbo Duan

Supplementary Information for

Evolutionary characteristics of global offshore carbon emissions network and responsibility allocation of emissions reduction

Bo Lu [1,*,#], Yue Sun [1], Lijie Fan [1], Xuejiao Ma [1,*], Hongbo Duan [2,*]

[1] School of Economics and Management, Dalian University of Technology, Dalian, 116024, China
[2] School of Economics and Management, University of Chinese Academy of Sciences, Beijing, 100190, China
* Correspondence: Email: lubo@dlut.edu.cn; maxuejiao@dlut.edu.cn; hbduan@ucas.ac.cn
[#] Lead Contact: Email: lubo@dlut.edu.cn

This PDF file includes:

Figure S1

Table S1-S4

Figure S1

Global offshore carbon emissions from 2015 to 2021 show a tendency of decrease firstly and then increase from 21112.58 Kt in 2015 to19360.38 Kt in 2018 to 20581.57 Kt in 2021 as shown in **Figure S1**. This may be because IMO has implemented the *Ship Energy Efficiency Management Plan* in 2014 to reduce energy efficiency, substitute traditional fuels by liquefied natural gas and alternative fuels and conduct operational mitigation measures. After 2018, the globalization accelerates the greenhouse gas emissions by promoting the continuous demands for sea transportation, causing the rise of carbon emissions. Therefore, there is a close relationship among the global trade, shipping and carbon emissions.

Carbon emissions from ports of top 20 countries contribute to about 80% of total port carbon emissions in the world, and top 20 countries are displayed in **Figure S1**. Among the 20 countries, there are 12 developed countries that generate 48% of port carbon emissions; while the other 8 developing countries generate about 52% of port carbon emissions. They are mainly located in Asia such as China and Singapore and Europe such as Italy and Spain. Specifically, China generates the largest carbon emissions from ports each year, followed by the U.S. and Singapore. As the biggest developing country, it has huge import and export markets, and the frequent seaborne trade is the root cause of its high port carbon emissions. The top 10 list of global port container throughput in 2021 includes 7 Chinese ports, and Shanghai port has always been in the top 1 position with nearly 10 million TEU more than the second port of Alphaliner. The industrial consumer goods of the U.S. are mainly dependent on imports, and its Log Angeles and Long Beach, with superior geography position, concentrate on external transportation, forming a commercial transportation system. So the intensive navigation of ships leads to the output of a very large amount of carbon emissions from ports. The Singapore Port has maintained its status as a global hub port and international maritime center. With the development of economic globalization, the increasing demands for shipping services have boosted the increase of carbon emissions from ports.

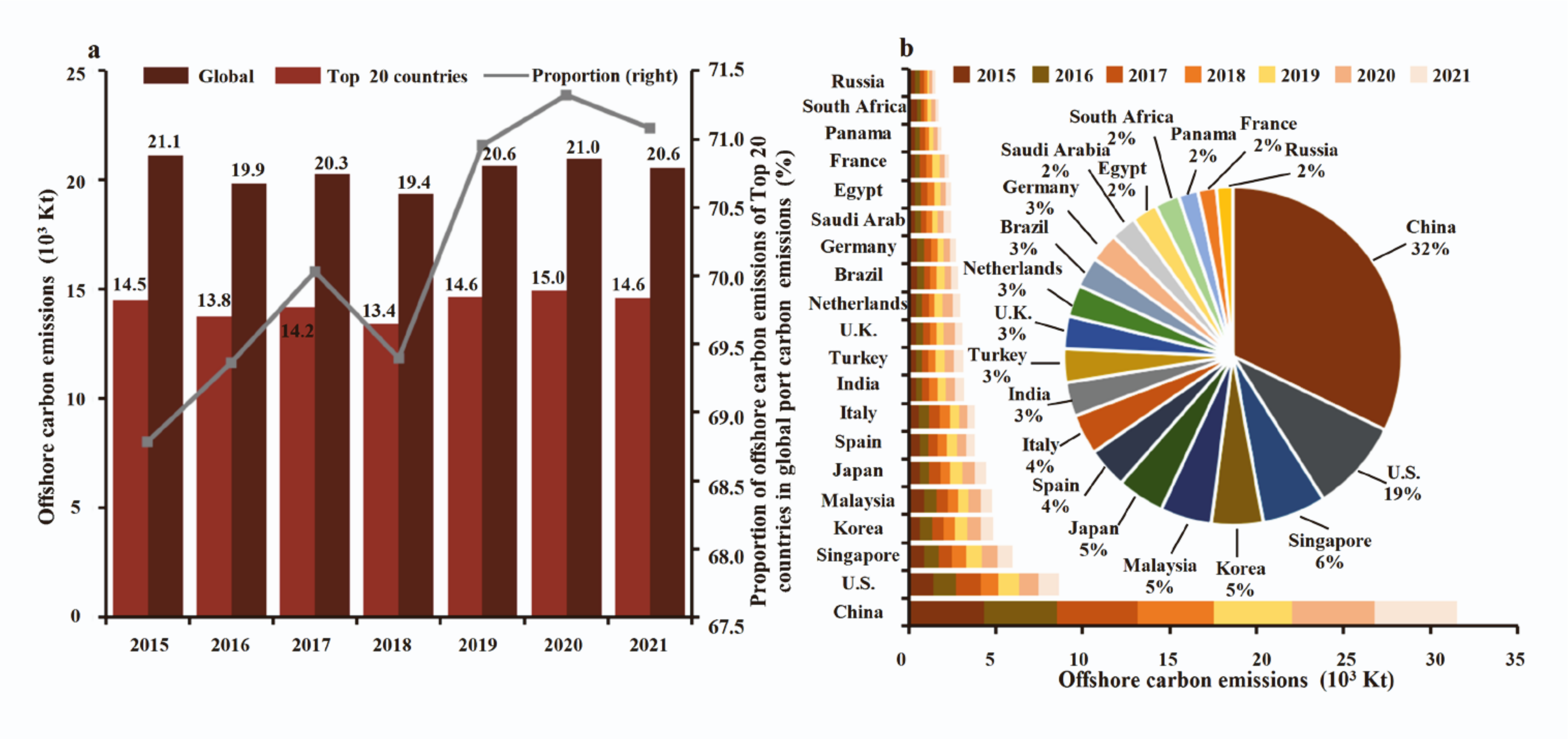

Figure S1. Offshore carbon emissions during 2015 and 2021

a, Total carbon emissions from global offshore and top 20 countries, and the proportion of offshore carbon emissions from top 20 offshore countries to global total offshore carbon emissions from 2015 to 2021. b, Evolution of offshore carbon emissions in top 20 countries during 2015 and 2021 (The pie chart shows the share of the average carbon emissions of top 20 countries from 2015 to 2021

Table S1. The developed countries and economies defined as the Annex I Parties of the Kyoto Protocol[1].

Table S1. The developed countries and economies defined as the Annex I Parties of the Kyoto Protocol.

**Developed countries and economies**		
United States of America	Germany	New Zealand
Australia	Greece	Norway
The Republic of Austria	Hungary	Poland
Belgium	Iceland	Portugal
Bulgaria	Ireland	Romania
Canada	Italy	Russian Federation
Croatia	Japan	The Slovak Republic
The Czech Republic	Latvia	Slovenia
Denmark	Liechtenstein	Spain
Estonia	Lithuania	Sweden
Finland	Luxembourg	Switzerland
European Community	Monaco	Ukraine
France	Netherlands	The United Kingdom of Great Britain and Northern Ireland

Table S2. Parameter values of engine’s installed power, average cruising speed and average maximum design speed for container ships with different load.

This table illustrates average propulsion engine’s installed power ($ME_{install,w}$), average cruising speed ($V_{man,w}$) and average maximum design speed ($V_{design,w}$) applied in this study for global container shipping emissions and energy consumption estimation.

Table S2. Parameter values of engine’s installed power, average cruising speed and average maximum design speed for container ships with different load

**Size category**	**2015** $ME_{install}$ **(kW)**	**2015** V_{design} **(kn)**	**2015** V_{man} **(kn)**	**2016** $ME_{install}$ **(kW)**	**2016** V_{design} **(kn)**	**2016** V_{man} **(kn)**	**2017** $ME_{install}$ **(kW)**	**2017** V_{design} **(kn)**	**2017** V_{man} **(kn)**	**2018-2020** $ME_{install}$ **(kW)**	**2018-2020** V_{design} **(kn)**	**2018-2020** V_{man} **(kn)**
0-999 TEU	5633	16.2	12.2	5509	16.1	12.2	5324	16.1	12.1	5077	16.0	11.8
1000-1999 TEU	12160	19.0	13.6	12119	19.0	13.7	12093	19.0	13.6	12083	19.0	13.4
2000-2999 TEU	21341	21.3	14.0	21010	21.2	14.1	20851	21.2	14.2	20630	21.1	14.2
3000-4999 TEU	35082	23.2	14.8	35133	23.2	14.8	34782	23.2	14.7	34559	23.1	14.7
5000-7999 TEU	52646	24.6	15.4	52597	24.6	15.5	52584	24.6	15.9	52566	24.6	15.7
8000-11999 TEU	59597	24.2	15.7	58665	24.0	16.1	58143	24.0	16.4	57901	23.9	16.3
12000-14499 TEU	65348	24	16.0	64498	23.9	16.3	62923	23.8	16.4	61231	23.8	16.3
14500-19999 TEU	61829	20.3	16.9	61417	20.0	17.1	60532	20.1	16.8	60202	20.2	16.5
20000-+ TEU	N/A	N/A	N/A	N/A	N/A	N/A	60681	20.4	15.2	60210	20.3	16.3

Table S3. Calculation of network characteristics

Table S3. Indicators of overall network and individual countries

Property	Name	Meaning	Equation	Practical implications	Value
Overall network	Network correlation	Direct or indirect path of connection between any two nodes	$NC = 1 - \frac{V}{N \times (N-1)/2}$	It reflects the stability of the network.	Higher value means a more stable network
	Network hierarchy	Asymmetrical reachability between nodes	$NH = 1 - \frac{K}{\max(K)}$	It reflects the dominant and hierarchical position of each country, reflecting the overall degree of asymmetry and whether each country plays an equal role.	Higher value means stricter hierarchical structure, and offshore carbon emissions between some countries are in a monopolic position.
	Network efficiency	Redundant connections between nodes	$NE = 1 - \frac{M}{\max(\mathrm{M})}$	It reflects the number of links between countries and channels for receiving and spilling carbon emissions.	Higher value means offshore carbon emissions have limited transfer channels.
Individual network	Degree centrality	The number of nodes directly related to a node	$DC(i) = \frac{n_{in} + n_{iout}}{2(N-1)}$	It reflects the status and control power of a country and whether a country is in a central position.	Higher value means important role and greater influence on other countries.
	Betweenness centrality	The number of the shortest paths of a node between any two other nodes	$C_B(i) = \frac{\sum_{j=1}^{N-1}\sum_{k=2}^{N} b_{jk}(i)}{(N^2 - 3N + 2)/2}$	It reflects the extent to which a country is able to act as an intermediary and the ability to control the transmission of information.	Higher value means stronger ability to control the relationship between other countries.

Note: V is the number of unreachable paired points in the network; N is the number of nodes in the network; $N \times (N-1)/2$ indicates the maximum number of possible connections in the network; K is the number of symmetrically reachable paired nodes in the network; max(K) is the maximum number of symmetrically reachable paired nodes in the network[2]; M is the number of extra

connections in the network; max(M) is the maximum number of possible redundant connections in the network; n_{in} represents the entry degree of the node (country) i; n_{iout} represents the output degree of the node (country) i; (n_{in}+n_{iout}) represents the number of countries directly connected to country i; $b_{jk}(i)$ represents the probability that country i lies between country j and k; $(N^2-3N+2)/2$ represents the maximum possible value of the betweenness centrality of a country in the case of star networks[3].

Table S4. Principal of block model

First, bidirectional spillover plate has many correlations with other plates and there are also close correlations between countries within the plate. Countries in this plate usually have the biggest ports in the world with close shipping trade with other countries, generating more carbon emissions. They play a leading role in the offshore carbon emissions network, and have a key driving effect on the other countries. Second, the broker plate is not closely connected with other plates, and connections between countries within it is few as well, which plays an intermediary role in the overall network. Countries in this plate can link different countries and ports may be in transit positions in global shipping trade. Third, in the main inflow plate, offshore carbon relationships between countries mainly occur within it, and there are less relationships with countries in other plates. Most of carbon emissions are from other plates, indicating that countries in this plate are the receivers of offshore carbon emissions. Fourth, in the main outflow plate, countries have more relationships with countries in other plates instead of countries within the plate. Most of the offshore carbon emissions of countries in this plate flow out and transfer to countries in other plates.

Table S4. Classification of offshore carbon emissions plate attributes of block model

Proportion of relations within plate	Proportion of relations received by plate	
	≈0	>0
$\geq (g-1)/(N-1)$	Bidirectional spillover plate	Main inflow plate
$< (g-1)/(N-1)$	Main outflow plate	Broker plate

Note: *g* is the number of members within the plate; *N* is the number of members in the whole network; (*g*-1)/(*n*-1) represents the expected internal relationship ratio. The total number of relationships received (sent) from (to) the plate is defined as the sum of the number of relationships for each column (row) in the received relationships matrix. Proportion of relations within the plate is the number of relationships in the plate divided by the total number of plate overflow relationships. Proportion of relations received by the plate is the number of relationships received from the other plates divided by the number of relationships sent to the other plates. When proportion of relations within plate is larger than the expected internal relationship ratio, it indicates that there is a closer correlation between the members within the plate. When proportion of relations received by plate is close to 0, indicating that there is a closer correlation between this plate and other plates.

References

1. Nations, U. (1998). Kyoto Protocol.
2. John Scott (2007). Social Network Analysis: A Handbook (Sage Publication).
3. Linton C. Freeman (1977). A Set of Measures of Centrality Based on Betweenness (Sociometry).
